# Supplementary material for: Phylogenomics of a new fungal phylum reveals multiple waves of reductive evolution across Holomycota
Source: Nat Commun. 2021 Aug 17;12:4973. doi: 10.1038/s41467-021-25308-w (PMC8371127; doi:10.1038/s41467-021-25308-w)
Supplement: Supplementary file 1 — Supplementary information [file 41467_2021_25308_MOESM1_ESM.pdf]

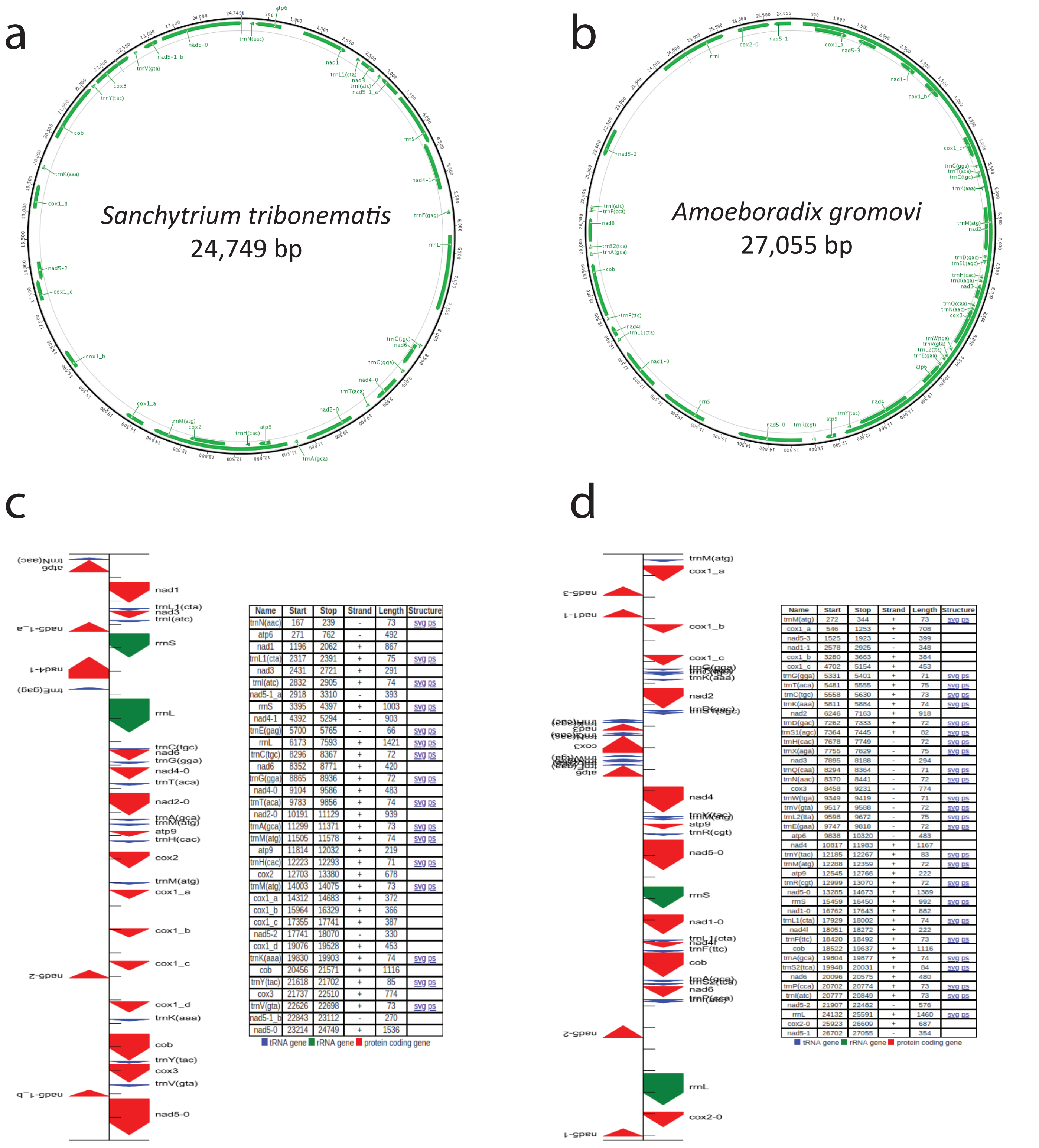

Name	Start	Stop	Strand	Length	Structure
trnN(aac)	167	239	-	73	svg ps
atp6	271	762	-	492	
nad1	1196	2062	+	867	
trnL1(cta)	2317	2391	+	75	svg ps
nad3	2431	2721	+	291	
trnI(atc)	2832	2905	+	74	svg ps
nad5-1_a	2918	3310	-	393	
rrnS	3395	4397	+	1003	svg ps
nad4-1	4392	5294	-	903	
trnE(gag)	5700	5765	-	66	svg ps
rrnL	6173	7593	+	1421	svg ps
trnC(tgc)	8296	8367	+	72	svg ps
nad6	8352	8771	+	420	
trnG(gga)	8865	8936	+	72	svg ps
nad4-0	9104	9586	+	483	
trnT(aca)	9783	9856	+	74	svg ps
nad2-0	10191	11129	+	939	
trnA(gca)	11299	11371	+	73	svg ps
trnM(atg)	11505	11578	+	74	svg ps
atp9	11814	12032	+	219	
trnH(cac)	12223	12293	+	71	svg ps
cox2	12703	13380	+	678	
trnM(atg)	14003	14075	+	73	svg ps
cox1_a	14312	14683	+	372	
cox1_b	15964	16329	+	366	
cox1_c	17355	17741	+	387	
nad5-2	17741	18070	-	330	
cox1_d	19076	19528	+	453	
trnK(aaa)	19830	19903	+	74	svg ps
cob	20456	21571	+	1116	
trnY(tac)	21618	21702	+	85	svg ps
cox3	21737	22510	+	774	
trnV(gta)	22626	22698	+	73	svg ps
nad5-1_b	22843	23112	-	270	
nad5-0	23214	24749	+	1536	

Name	Start	Stop	Strand	Length	Structure
trnM(atg)	272	344	+	73	svg ps
cox1_a	546	1253	+	708	
nad5-3	1525	1923	-	399	
nad1-1	2578	2925	-	348	
cox1_b	3280	3663	+	384	
cox1_c	4702	5154	+	453	
trnG(gga)	5331	5401	+	71	svg ps
trnT(aca)	5481	5555	+	75	svg ps
trnC(tgc)	5558	5630	+	73	svg ps
trnK(aaa)	5811	5884	+	74	svg ps
nad2	6246	7163	+	918	
trnD(gac)	7262	7333	+	72	svg ps
trnS1(agc)	7364	7445	+	82	svg ps
trnH(cac)	7678	7749	-	72	svg ps
trnX(aga)	7755	7829	-	75	svg ps
nad3	7895	8188	-	294	
trnQ(caa)	8294	8364	-	71	svg ps
trnN(aac)	8370	8441	-	72	svg ps
cox3	8458	9231	-	774	
trnW(tga)	9349	9419	-	71	svg ps
trnV(gta)	9517	9588	-	72	svg ps
trnL2(tta)	9598	9672	-	75	svg ps
trnE(gaa)	9747	9818	-	72	svg ps
atp6	9838	10320	-	483	
nad4	10817	11983	+	1167	
trnY(tac)	12185	12267	+	83	svg ps
trnM(atg)	12288	12359	+	72	svg ps
atp9	12545	12766	+	222	
trnR(cgt)	12999	13070	+	72	svg ps
nad5-0	13285	14673	+	1389	
rrnS	15459	16450	+	992	svg ps
nad1-0	16762	17643	+	882	
trnL1(cta)	17929	18002	+	74	svg ps
nad4l	18051	18272	+	222	
trnF(ttc)	18420	18492	+	73	svg ps
cob	18522	19637	+	1116	
trnA(gca)	19804	19877	+	74	svg ps
trnS2(tca)	19948	20031	+	84	svg ps
nad6	20096	20575	+	480	
trnP(cca)	20702	20774	+	73	svg ps
trnI(atc)	20777	20849	+	73	svg ps
nad5-2	21907	22482	-	576	
rrnL	24132	25591	+	1460	svg ps
cox2-0	25923	26609	+	687	
nad5-1	26702	27055	-	354	

Supplementary Fig. 1. MITOS Graphical representation of the mitochondrial genome and gene content of *Sanchytrium tribonematis* (**a** and **c**), and *Amoeboradix gromovi* (**b** and **d**).

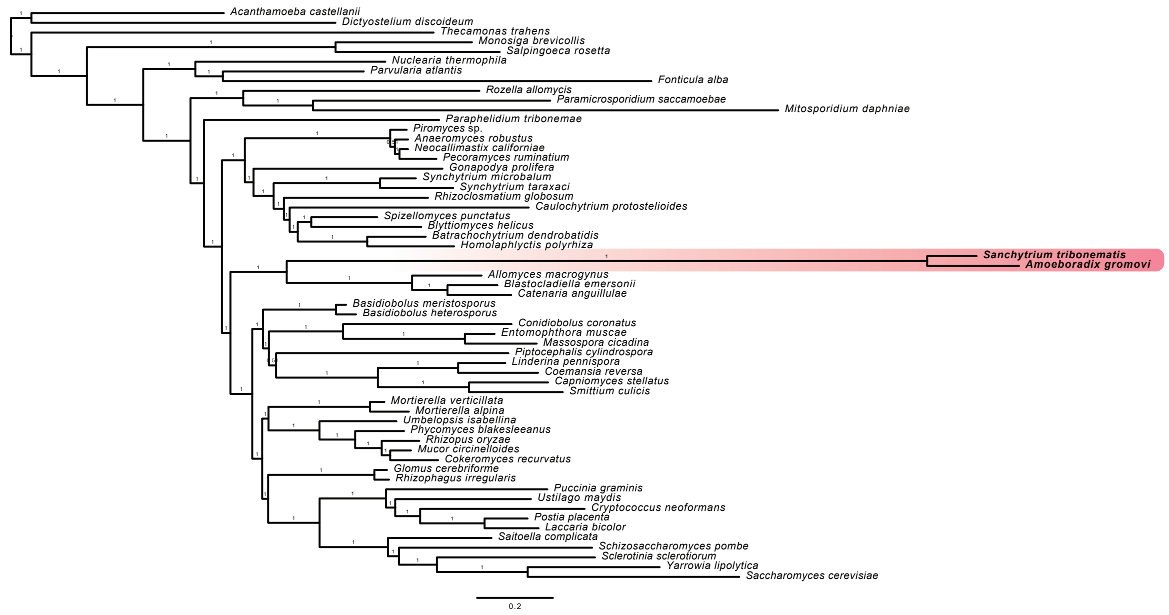

Supplementary Fig. 2. a, Bayesian phylogenomic tree based on the GBE protein dataset. The tree was reconstructed using 264 conserved proteins, 59 species, and 93,743 conserved amino acid positions, it was inferred using PhyloBayes under the CAT-Poisson model with posterior probability as statistical support.

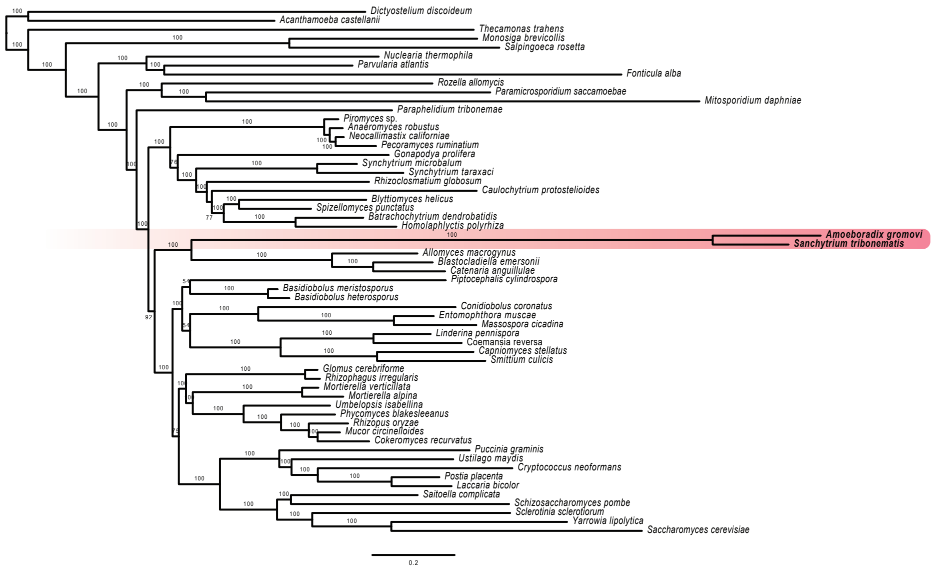

Supplementary Fig. 2. b, Maximum likelihood tree based on the GBE protein dataset. The tree was reconstructed using 264 conserved proteins, 59 species, and 93,743 conserved amino acid positions, it was inferred with IQ-TREE under the PMSF approximation of the LG+R8+C20 model and ultrafast bootstrap as statistical support.

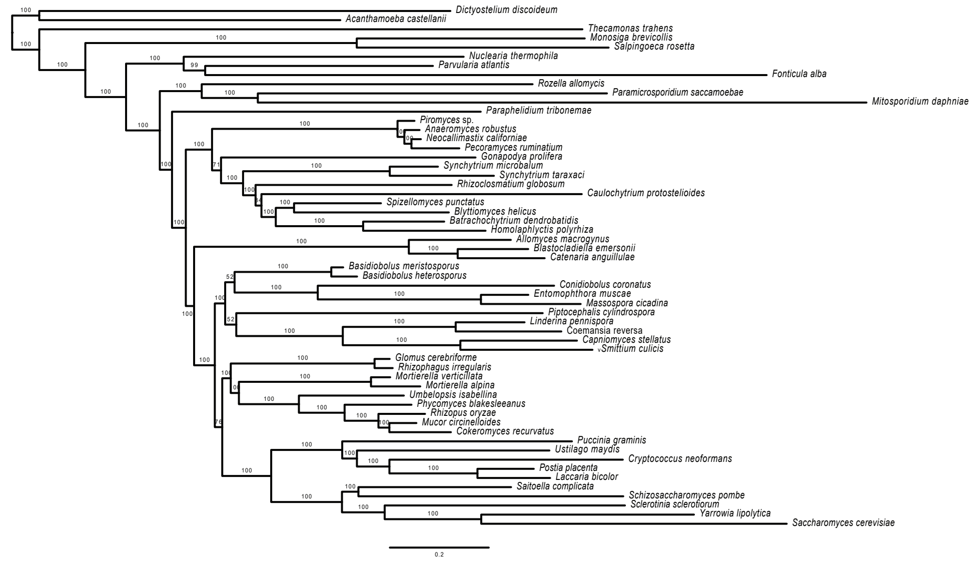

Supplementary Fig. 2. c, Maximum likelihood tree based on the GBE protein dataset. The tree was reconstructed using 264 conserved proteins, 57 species, and 93,421 conserved amino acid positions, it was inferred with IQ-TREE under the PMSF approximation of the LG+R8+C20 model and ultrafast bootstrap as statistical support.

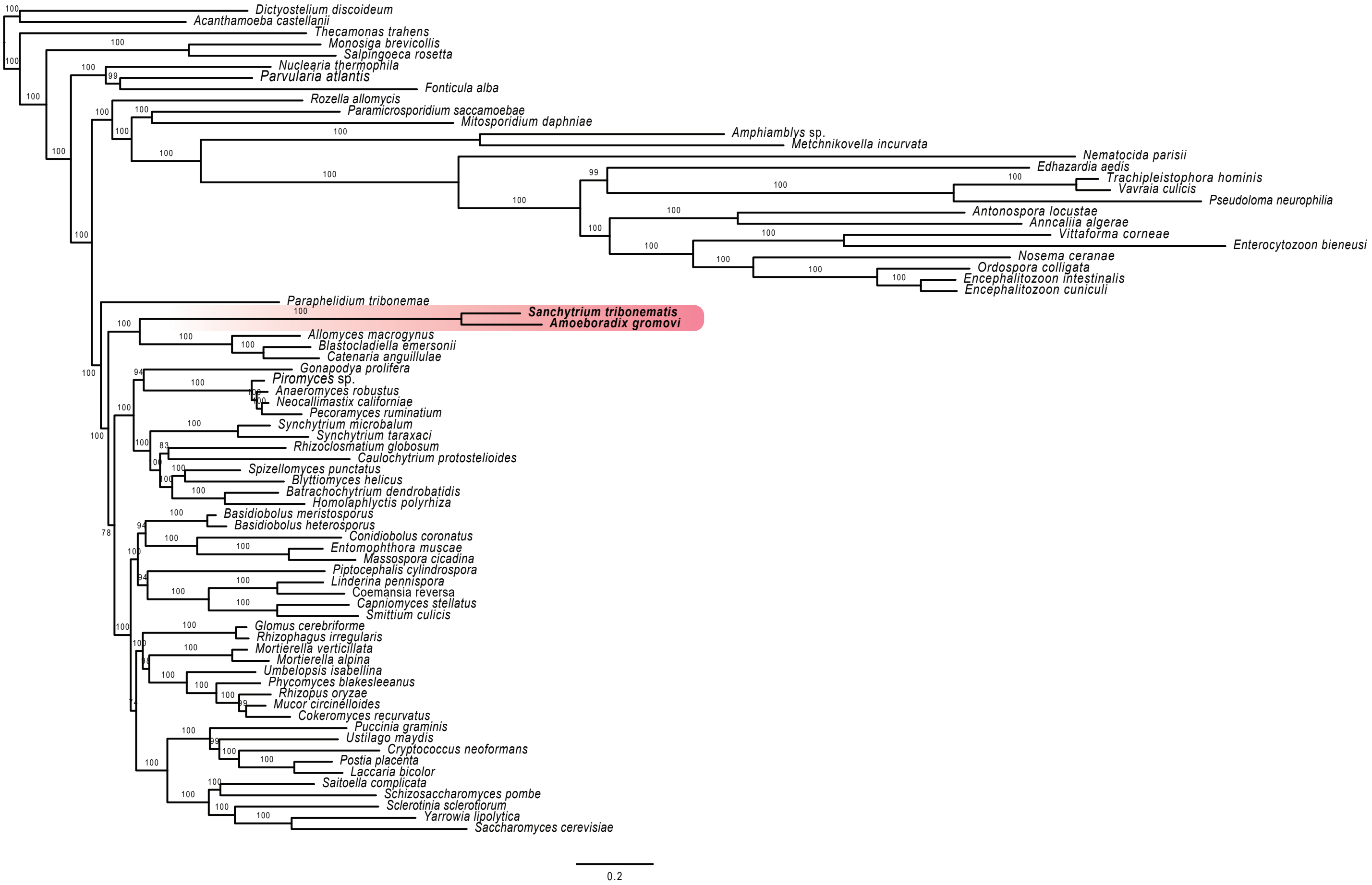

Supplementary Fig. 2. d, Maximum likelihood tree based on the GBE protein dataset. The tree was reconstructed using 264 conserved proteins, 74 species, and 86,313 conserved amino acid positions, it was inferred with IQ-TREE under the PMSF approximation LG+F+R10+C20 model and ultrafast bootstrap as statistical support.

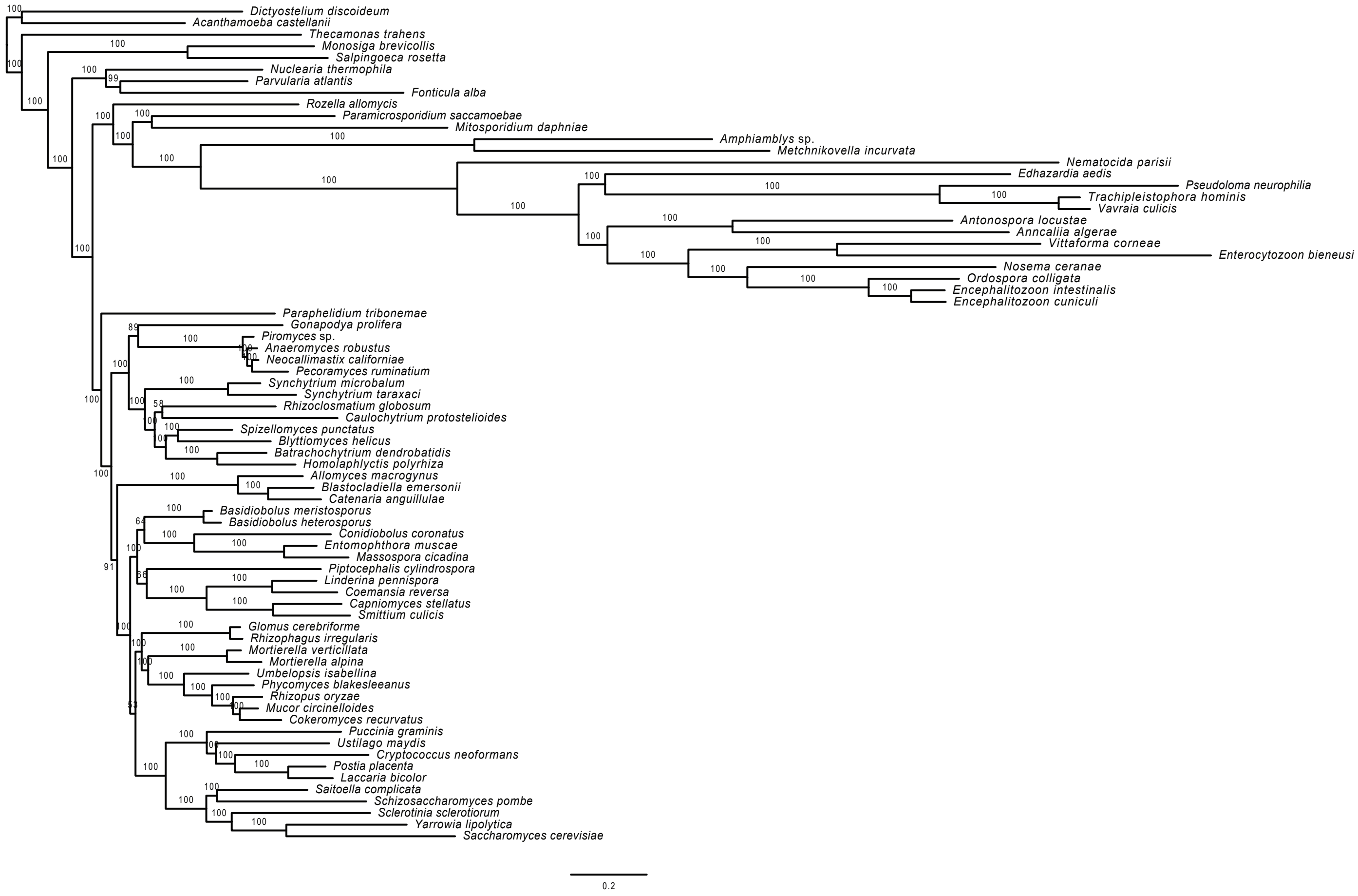

Supplementary Fig. 2. e, Maximum likelihood tree based on the GBE protein dataset. The tree was reconstructed using 264 conserved proteins, 72 species, and 84,949 conserved amino acid positions, it was inferred with IQ-TREE under the PMSF approximation LG+F+R9+C20 model and ultrafast bootstrap as statistical support.

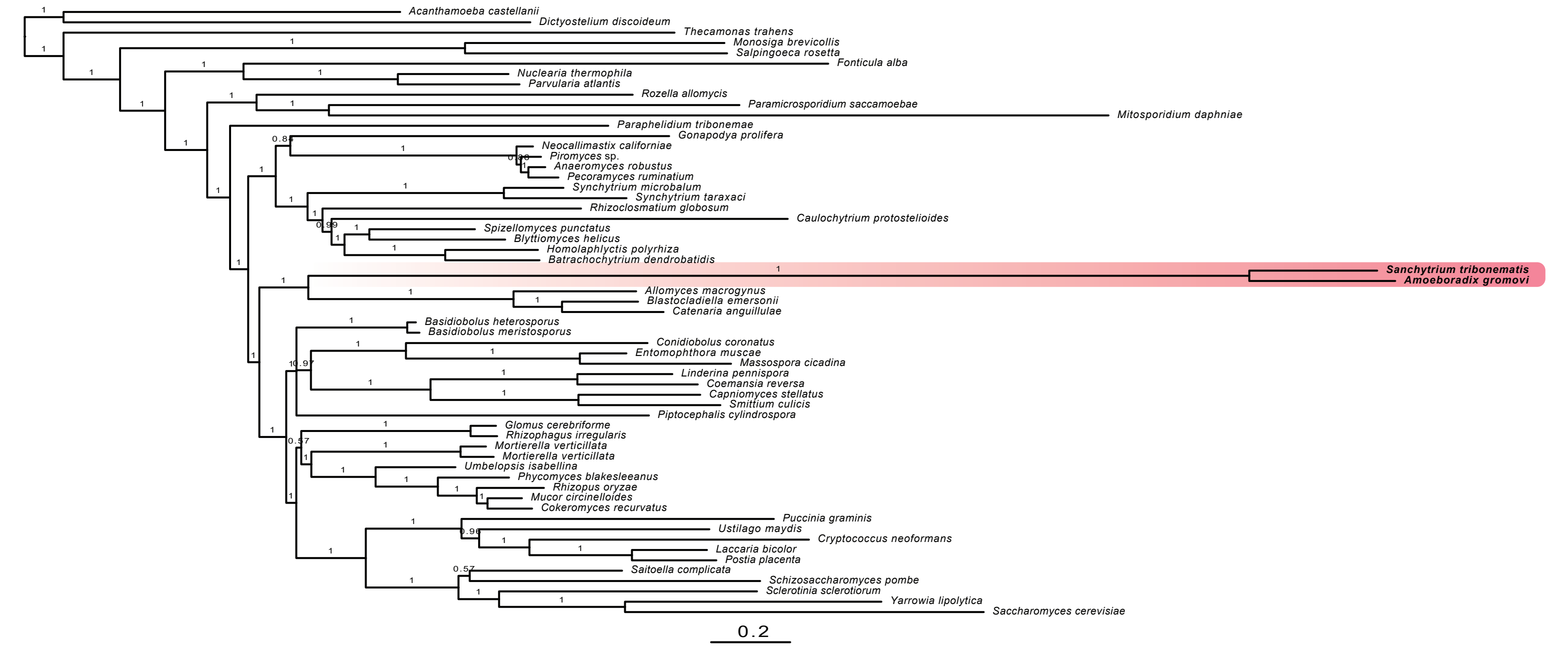

Supplementary Fig. 2. f, Bayesian phylogenomic tree based on the BMC protein dataset. The tree was reconstructed using 53 conserved proteins, 59 species, and 14,965 conserved amino acid positions, it was inferred using PhyloBayes under the CAT-GTR model with posterior probability as statistical support.

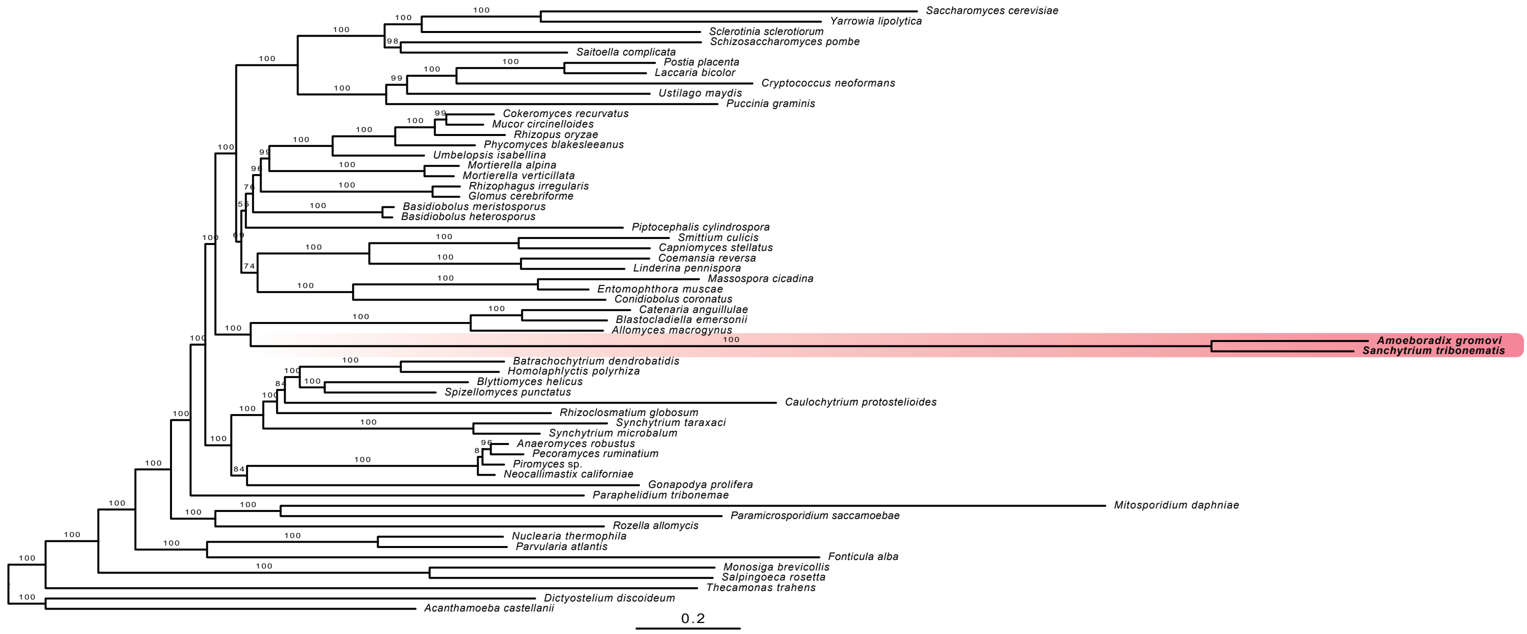

Supplementary Fig. 2. g, Maximum likelihood tree based on the BMC protein dataset. The tree was reconstructed using 53 conserved proteins, 59 species, and 14,965 conserved amino acid positions, it was inferred with IQ-TREE under the PMSF approximation of the LG+R7+C60 model and ultrafast bootstrap as statistical support.

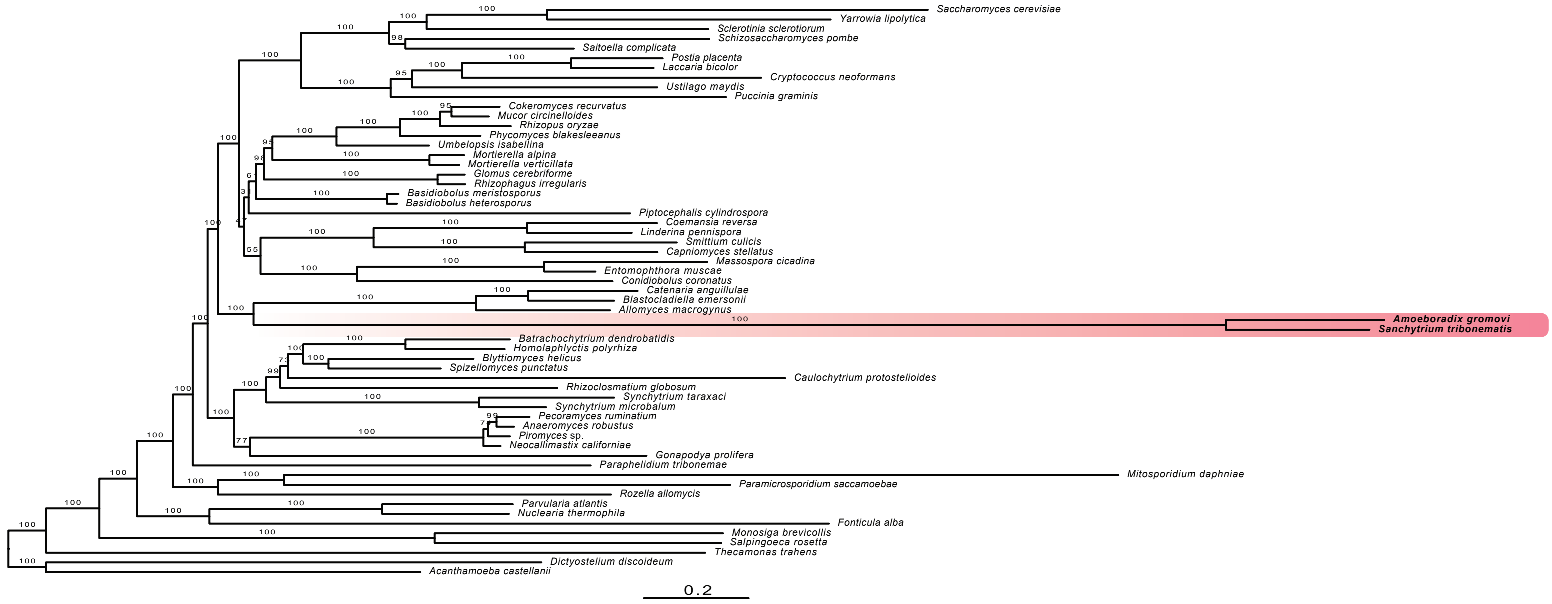

Supplementary Fig. 2. h, Maximum likelihood tree based on the BMC protein dataset. The tree was reconstructed using 53 conserved proteins, 59 species, and 14,965 conserved amino acid positions, it was inferred with IQ-TREE under the PMSF approximation of the LG+R7+C60 model and 100 conventional bootstrap replicates as statistical support.

Gene	*Cyberlindnera jadinii*	*Saccharomyces cerevisiae*	*Hanseniaspora gamundiae*	*Hanseniaspora singularis*	*Allomyces macrogynus*	***Amoeboradix gromovi***	***Sanchytrium tribonematis***
Sen15	Present	Present	Present	Absent	Absent	Absent	Absent
Mcm21	Present	Present	Present	Absent	Absent	Absent	Absent
Sld2	Present	Present	Present	Absent	Absent	Absent	Absent
Cdc13	Present	Present	Present	Absent	Absent	Absent	Absent
Mcm22	Absent	Present	Present	Absent	Absent	Absent	Absent
Abf1	Absent	Present	Present	Absent	Absent	Absent	Absent
Rfa3	Absent	Present	Present	Absent	Absent	Absent	Absent
Csm2	Absent	Present	Present	Absent	Absent	Absent	Absent
Fyv6	Present	Present	Absent	Absent	Absent	Absent	Absent
Rif1	Present	Present	Present	Absent	Absent	Absent	Absent
Pds1	Present	Present	Present	Absent	Absent	Absent	Absent
Mms22	Present	Present	Present	Absent	Absent	Absent	Absent
Eco1	Present	Present	Present	Absent	Present	Absent	Absent
Mms4	Present	Present	Present	Absent	Absent	Absent	Absent
Mrc1	Present	Present	Present	Absent	Absent	Absent	Absent
Nse1	Present	Present	Present	Absent	Absent	Absent	Absent
Nse3	Present	Present	Present	Absent	Absent	Absent	Absent
Nse5	Present	Present	Present	Absent	Absent	Absent	Absent
Nup120	Present	Present	Present	Absent	Absent	Absent	Absent
Nup133	Present	Present	Present	Absent	Absent	Absent	Absent
Nup60	Present	Present	Present	Absent	Absent	Absent	Absent
Rad9	Present	Present	Present	Absent	Absent	Absent	Absent
Slx4	Present	Present	Present	Absent	Absent	Absent	Absent
Snf6	Present	Present	Present	Absent	Absent	Absent	Absent
Tah11	Present	Present	Present	Absent	Absent	Absent	Absent
Eaf6	Absent	Present	Present	Absent	Present	Absent	Absent
Pol32	Present	Present	Present	Absent	Absent	Absent	Absent
Def1	Present	Present	Present	Absent	Absent	Absent	Absent
Tdp1	Present	Present	Present	Absent	Absent	Present	Absent
Pol4	Present	Present	Present	Absent	Present	Present	Present
Psy3	Present	Present	Absent	Absent	Absent	Absent	Absent
Hpr1	Present	Present	Absent	Absent	Absent	Present	Present
Sae3	Present	Present	Absent	Absent	Absent	Absent	Absent
Kre29	Absent	Present	Absent	Absent	Absent	Absent	Absent
Eaf5	Absent	Present	Absent	Absent	Absent	Absent	Absent
Mms1	Absent	Present	Absent	Absent	Absent	Absent	Absent
Nej1	Absent	Present	Absent	Absent	Absent	Absent	Absent
Sir4	Absent	Present	Absent	Absent	Absent	Absent	Absent
Lif1	Absent	Present	Absent	Absent	Absent	Absent	Absent
Xrs2	Absent	Present	Absent	Absent	Absent	Absent	Absent
Eaf7	Absent	Present	Absent	Absent	Present	Absent	Absent
Mag1	Present	Present	Absent	Absent	Absent	Absent	Absent
Eaf3	Present	Present	Absent	Absent	Present	Present	Present
Pcd1	Present	Present	Absent	Absent	Present	Present	Present
Phr1	Present	Present	Absent	Absent	Present	Present	Present
Mgt1	Absent	Present	Absent	Present	Present	Present	Present
Lrs4	Absent	Present	Present	Absent	Absent	Absent	Absent

Present
Absent

Supplementary Fig. 3. Genome maintenance and DNA repair genes in *Amoeboradix* and *Sanchytrium*, compared with a close relative (the blastoclad *Allomyces*) and several slow- and fast-evolving yeasts. Based on the study by Steenwyk et al. (2019) Extensive loss of cell-cycle and DNA repair genes in an ancient lineage of bipolar budding yeasts. PLoS Biol 17(5): e3000255.

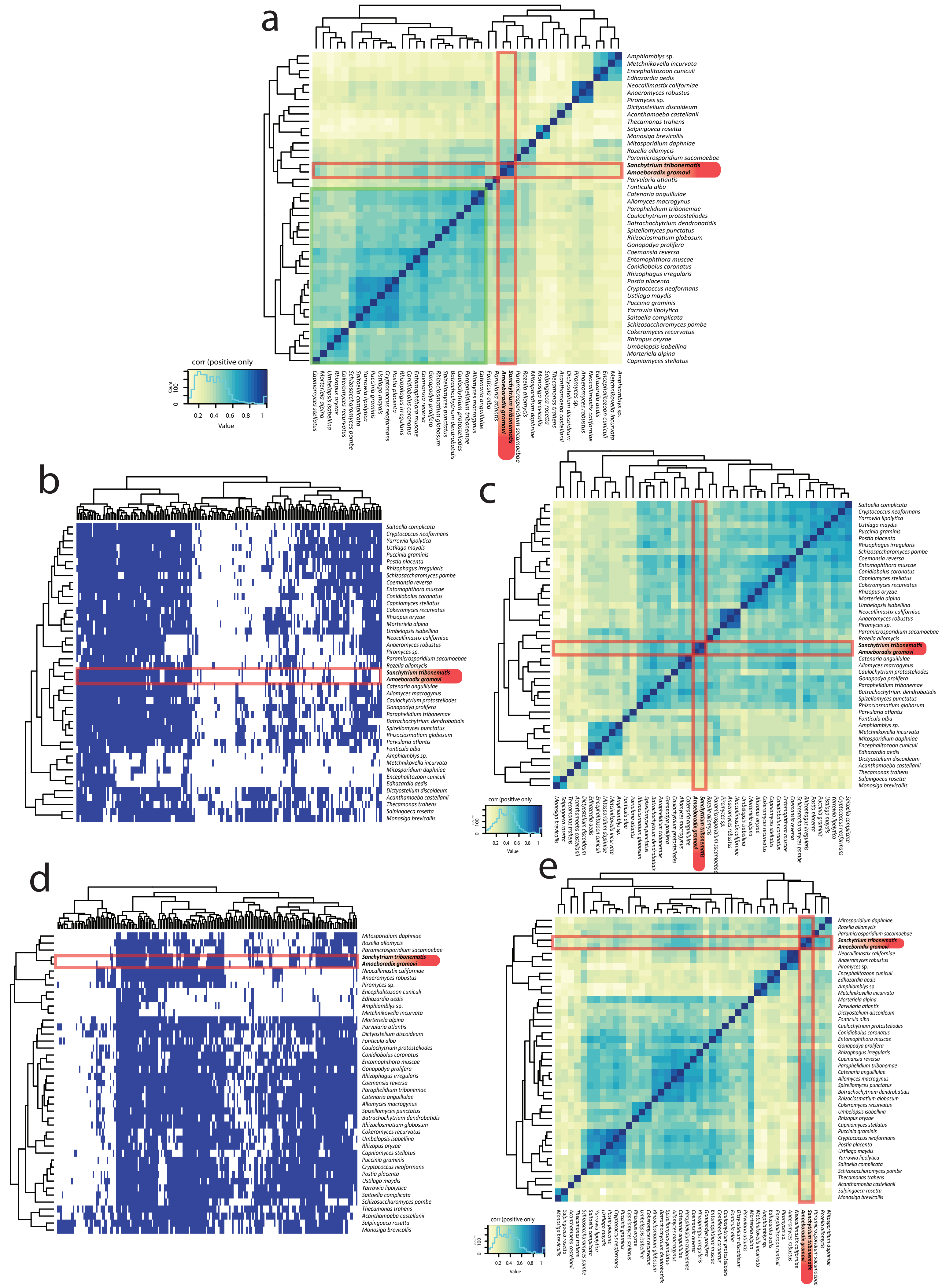

Supplementary Fig. 4. Comparative analyses of primary metabolism. a, Clustering of 43 eukaryotic species according to their profile of presence/absence of the same 8 primary metabolism categories for a total of 1158 COGs. Color code is scaled to display positive Pearson's r (0 to 1). (**b** and **d**) Binary heat maps of orthologs (COGs) in 43 eukaryotic genomes/transcriptomes. (**c** and **e**) Clustering of 43 eukaryotic genomes/transcriptomes according to their profile of presence/absence color coded to display positive Pearson's r (0 to 1) (**b** and **c**) Carbohydrate transport and metabolism (205 OGs, category G). (**d** and **e**) Lipid transport and metabolism (201 OGs, category I).

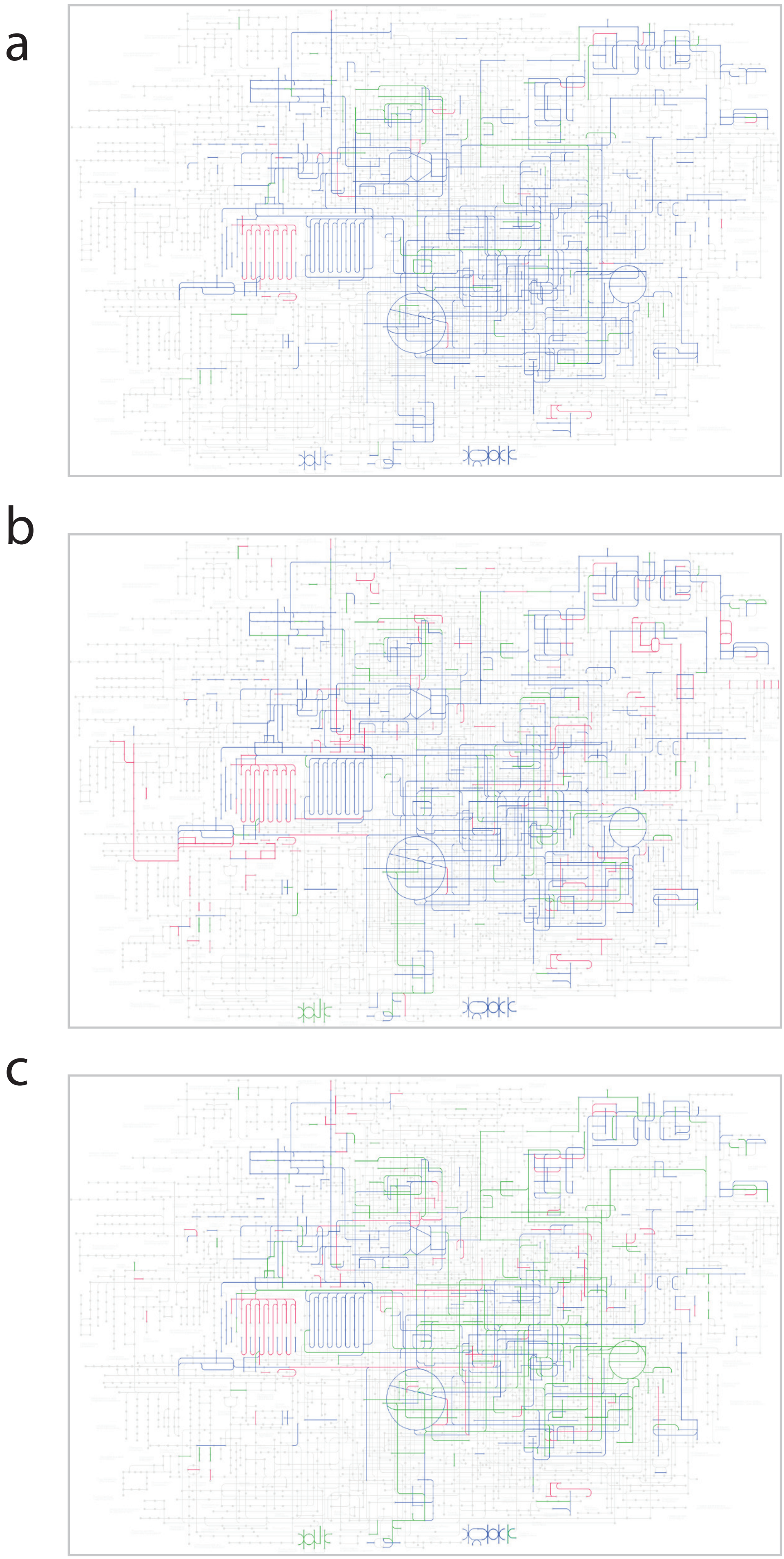

Supplemetary Fig. 5. a - c, KEGG Pathways metabolic comparison. (**a** - **c**) KEGG metabolic pathways map01100. **a**, *Sanchytrium tribonematis* (green) vs *Amoeboradix gromovi* (pink); both (blue). **b**, *Sanchytrium tribonematis* (green) vs *Allomyces macrogynus* (pink); both (blue). **c**, *Sanchytrium tribonematis* (green) vs *Rozella allomycis* (pink); both (blue).

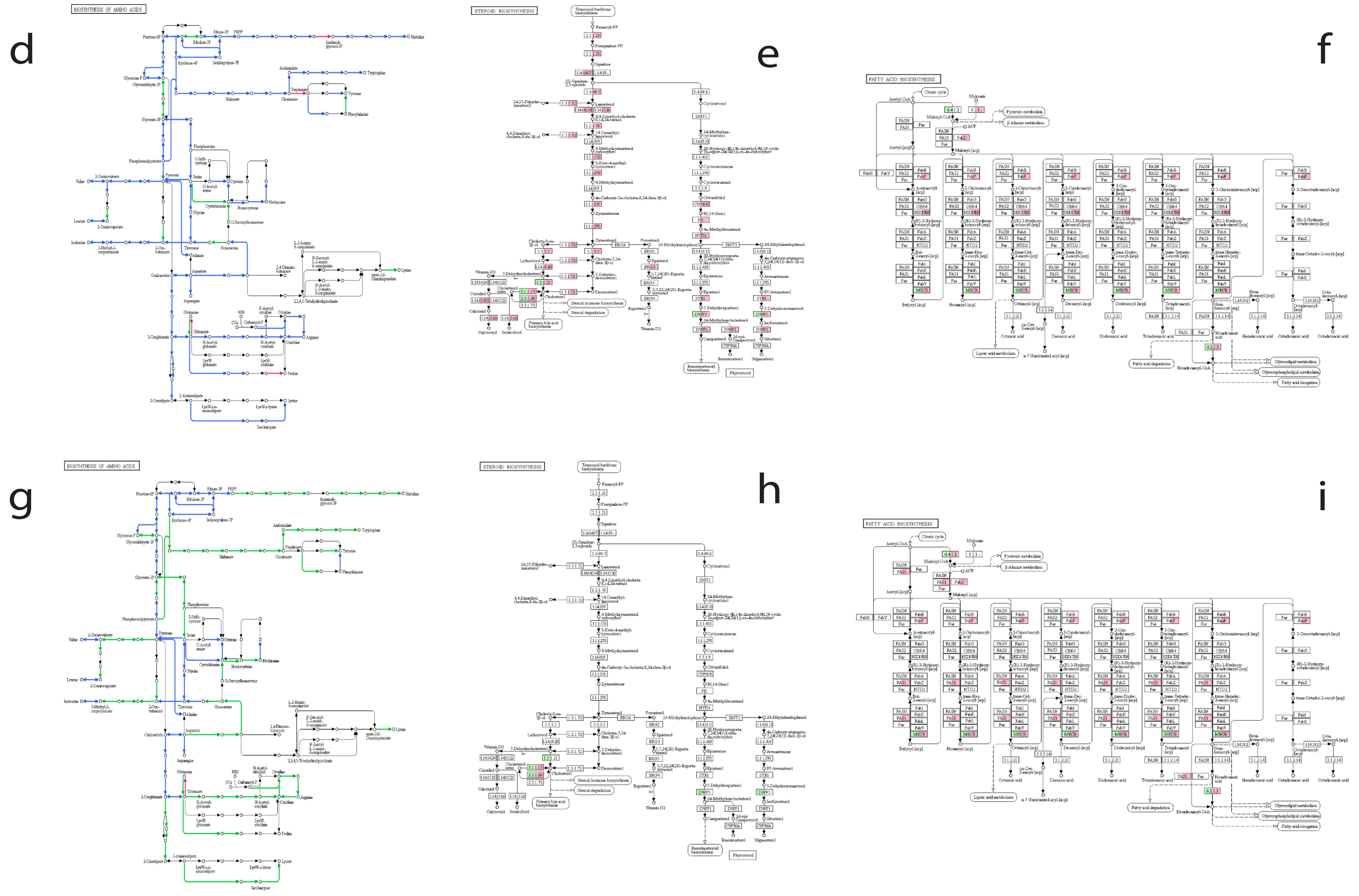

Supplementary Fig. 5. d -i, KEGG Pathways metabolic comparison. (**d** - **f**) KEEG specific metabolic pathways of *Sanchytrium tribonematis* (green) vs *Allomyces macrogynus* (pink); both (blue). (**g** - **i**) KEEG specific metabolic pathways of *Sanchytrium tribonematis* (green) vs *Rozella allomycis* (pink); both (blue). (**d** and **g**) KEGG Amino acid synthesis map01230. (**e** and **h**) KEGG Steroid biosynthesis map00100. (**f** and **i**) KEGG Fa y acid biosynthesis map00061.

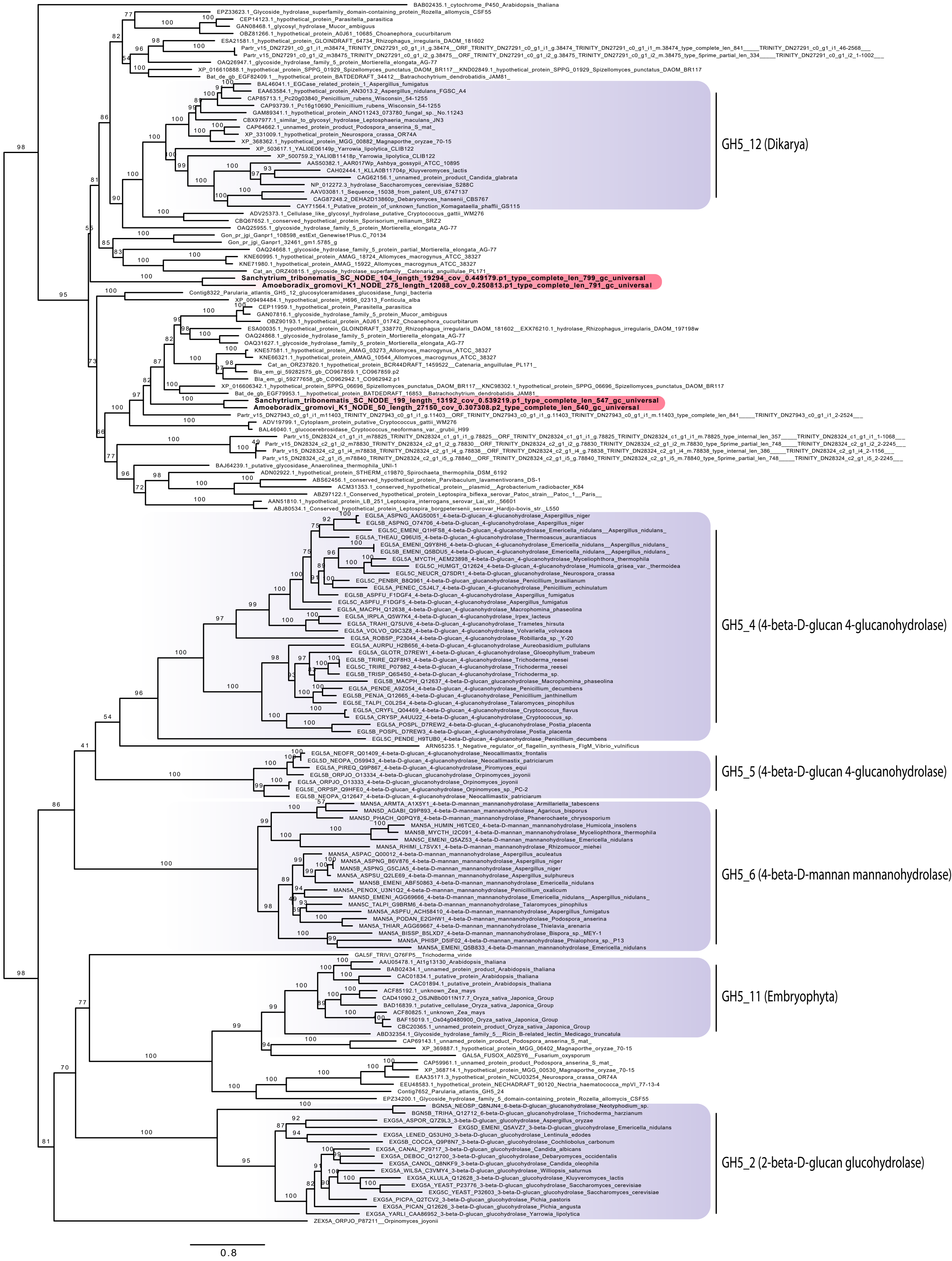

Supplementary Fig. 6. Maximum likelihood tree of the GH5 cellulase family. The tree was reconstructed based on the dataset of Torruella et al. 2018, 170 sequences and 4,721 amino acid positions with the LG+R7 model for ML. Ultrafast bootstrap (ufbs) support values are indicated over the branches.

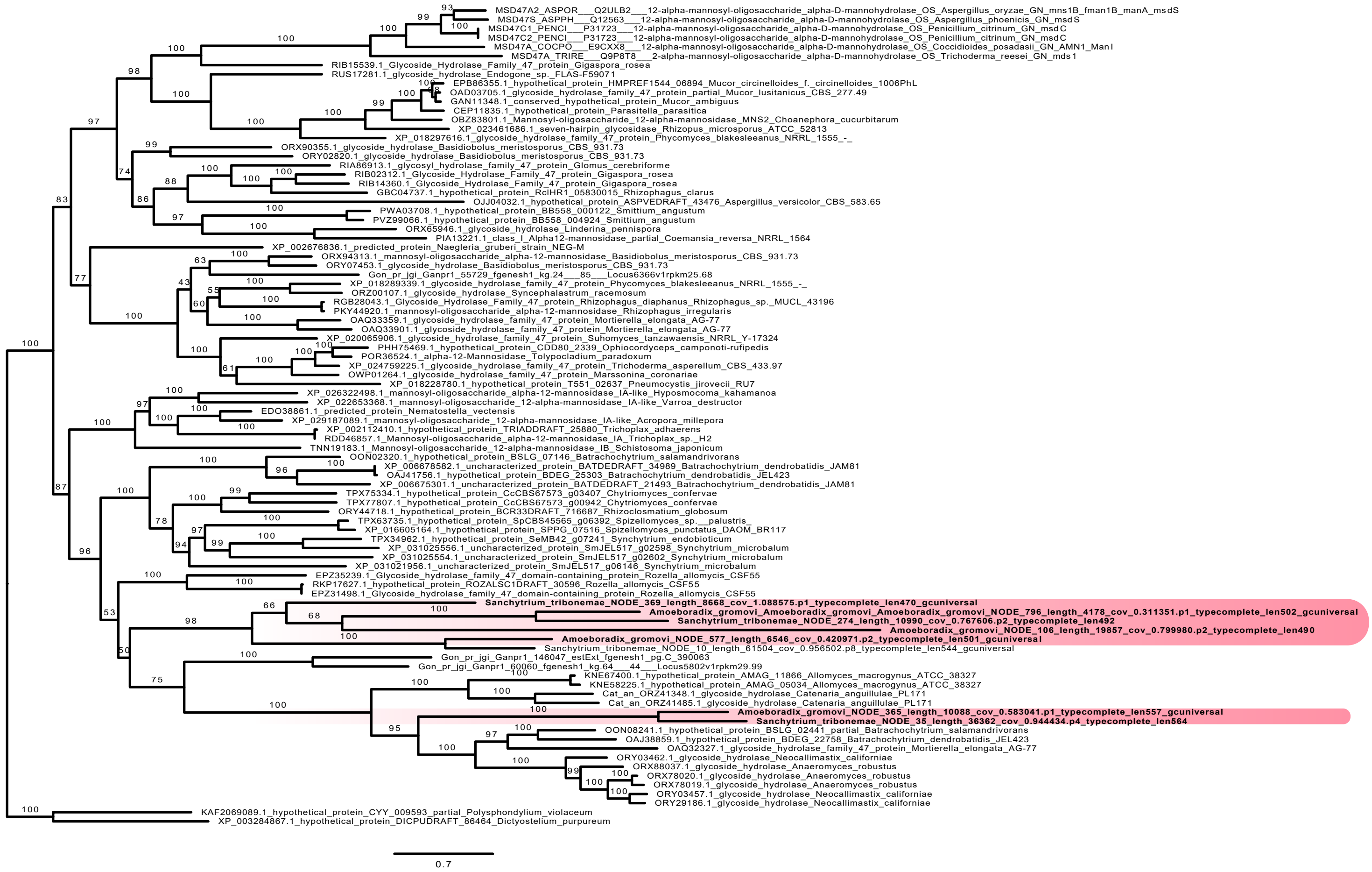

Supplementary Fig. 7. Maximum likelihood tree of the GH47 alpha-1,2-mannosidase family of hemicellulose degradation, containing a total of 90 sequences and 1,910 amino acid positions with the LG+F+R6 model for ML. Ultrafast bootstrap (ufbs) support values are indicated over the branches.

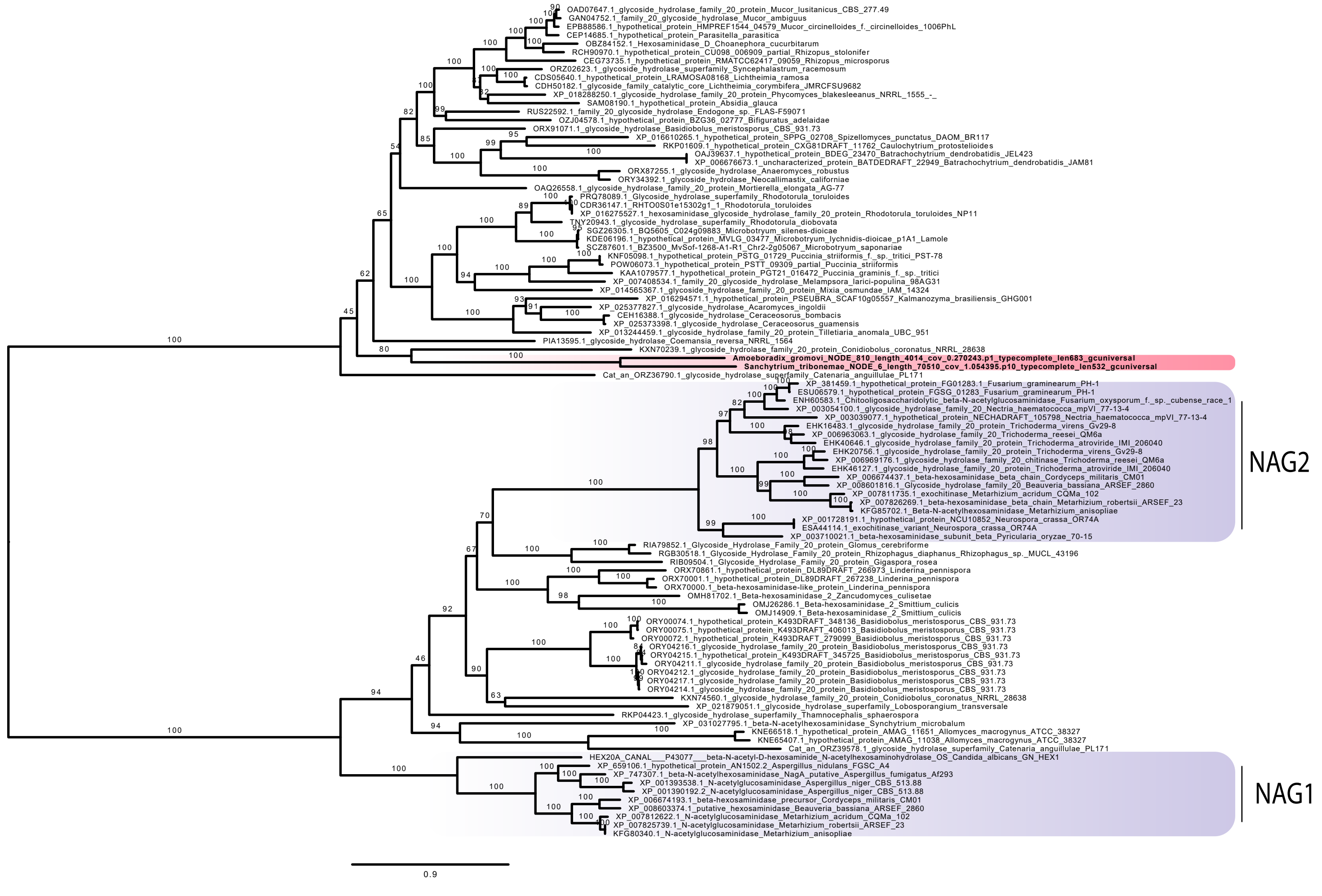

Supplementary Fig. 8. Maximum likelihood tree of family GH20 b-N-acetylhexosaminidases (NAGase) containing a total of 98 sequences and 2,031 amino acid positions with the LG+F+R7 model for ML. The tree was reconstructed based on the dataset of de Oliveira et al. 2018. Ultrafast bootstrap (ufbs) support values are indicated over the branches.

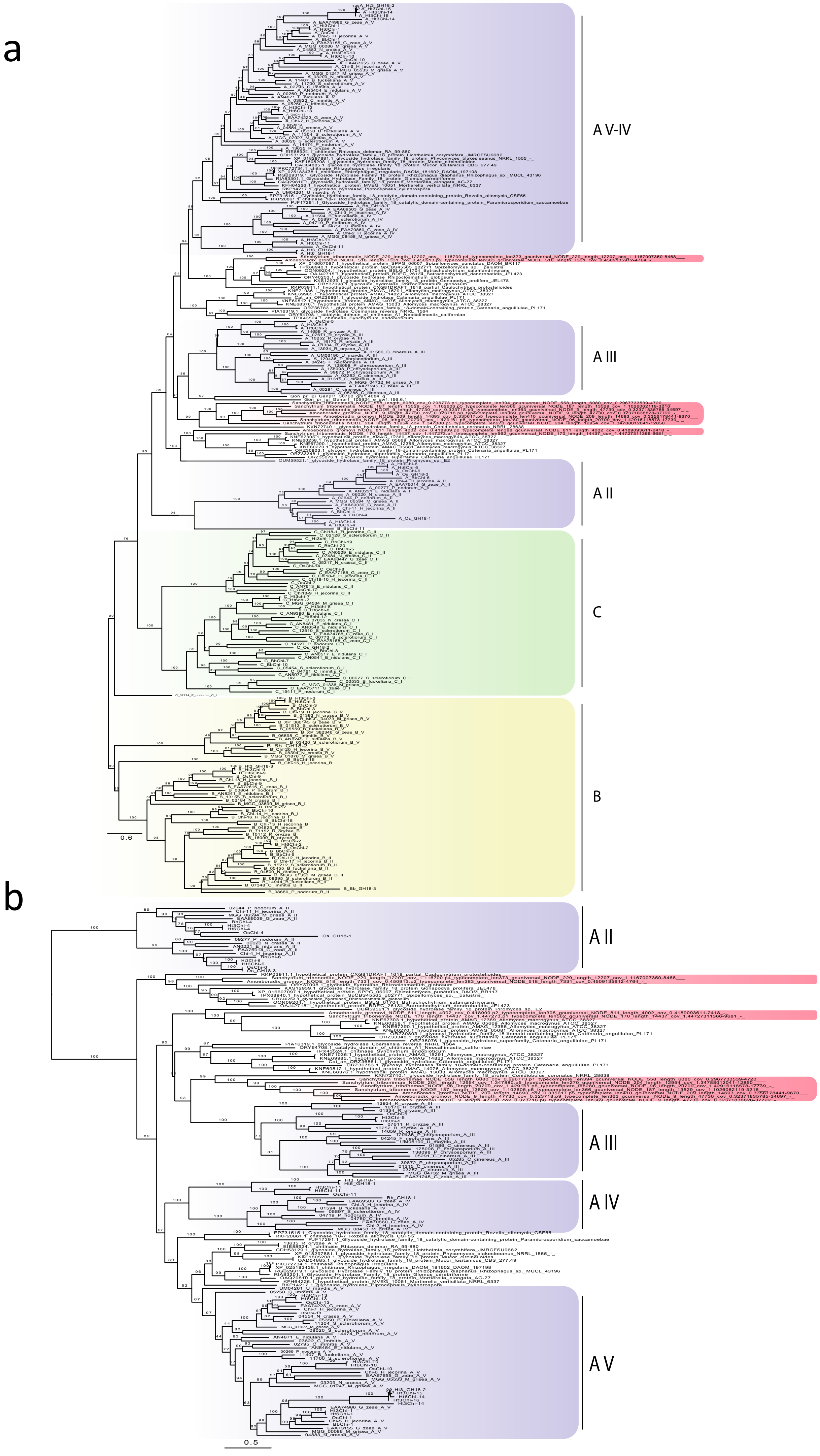

Supplementary Fig. 9. Maximum likelihood trees of the GH18 chytinase family. The trees were reconstructed based on the dataset of Agrawal et al. 2015. (A) Maximum likelihood tree of 262 sequences and 5,761 amino acid positions with the LG+R8 model for ML. (B) Maximum likelihood tree of family GH18A of 152 sequences and 2,746 amino acid positions with the LG+R6 model for ML. Ultrafast bootstrap (ufbs) support values are indicated over the branches.

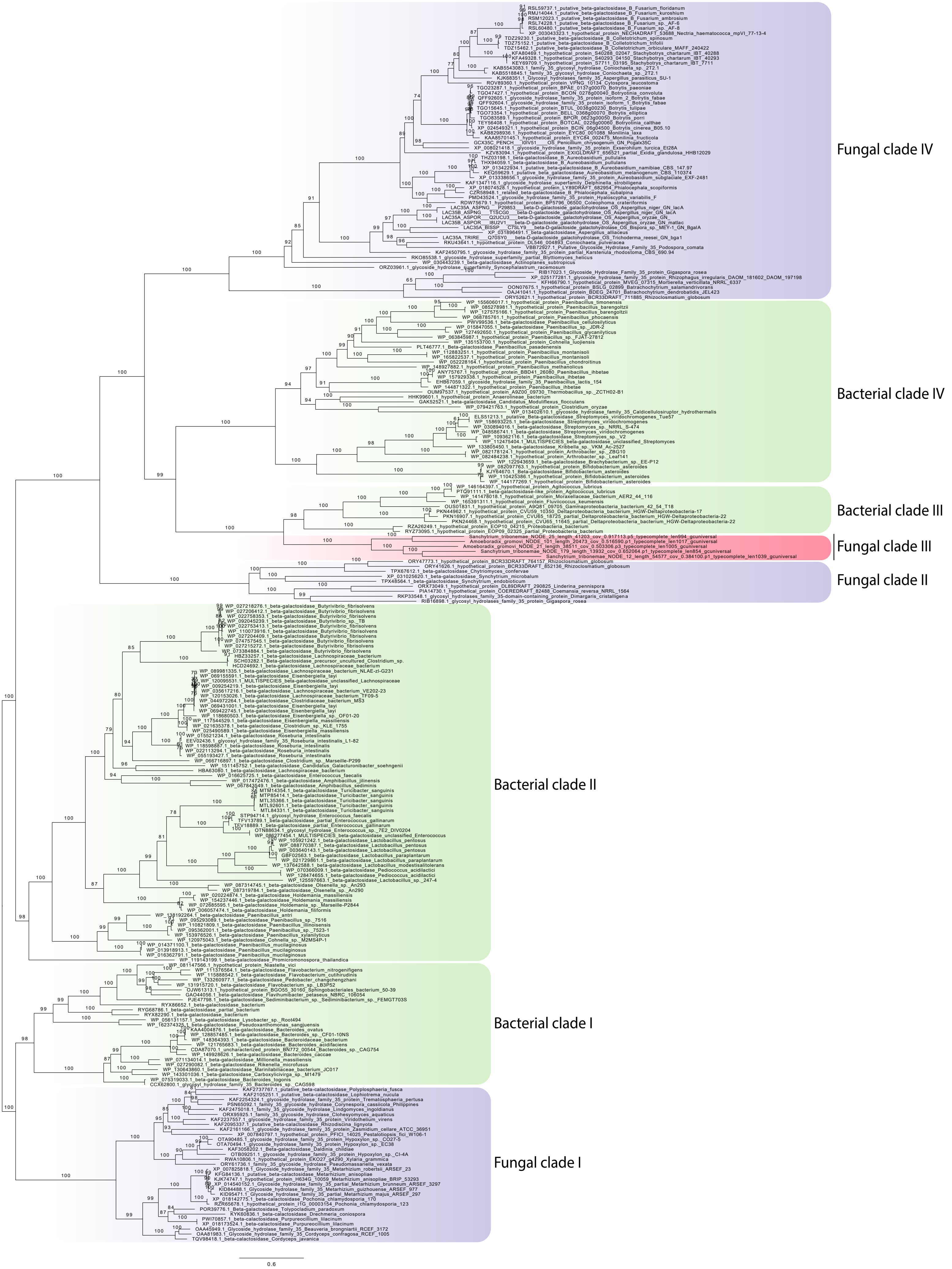

Supplementary Fig. 10. Maximum likelihood tree of the GH35 β-galactosidase family. Maximum likelihood tree of 248 sequences and 2,424 amino acid positions with the LG+R7 model for ML. Ultrafast bootstrap (ufbs) support values are indicated over the branches.

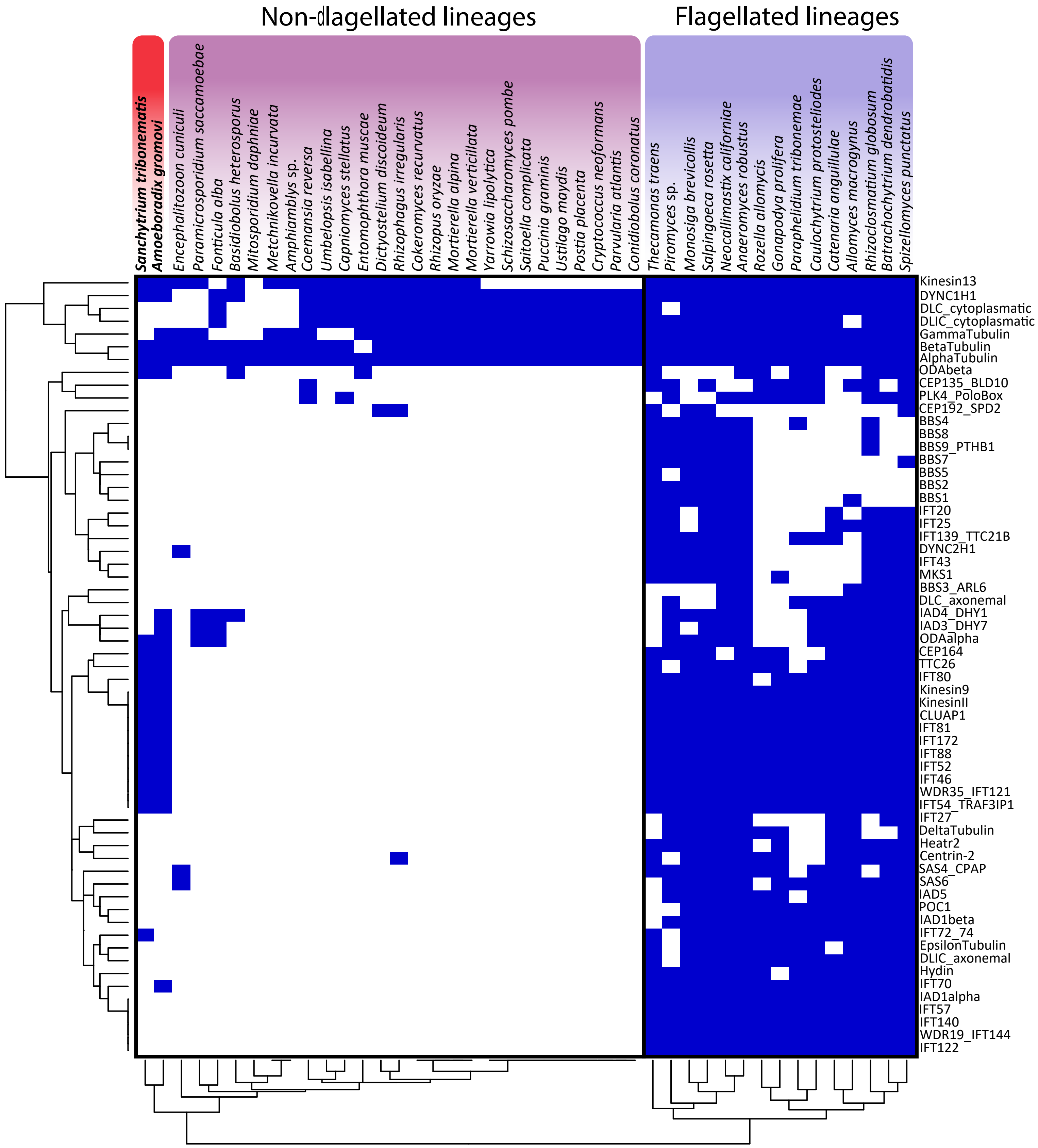

Supplementary Fig. 11. Flagellum toolkit reduction. Presence/absence heatmaps of 61 flagellum-specific proteins in 43 eukaryotic flagellated (purple) and non-flagellated (pink) lineages. Heatmap clustered by similarity showing sanchytrids in an intermediate position between flagellated and non-flagellated lineages. Gene presence is depicted in blue and absence is depicted in white.

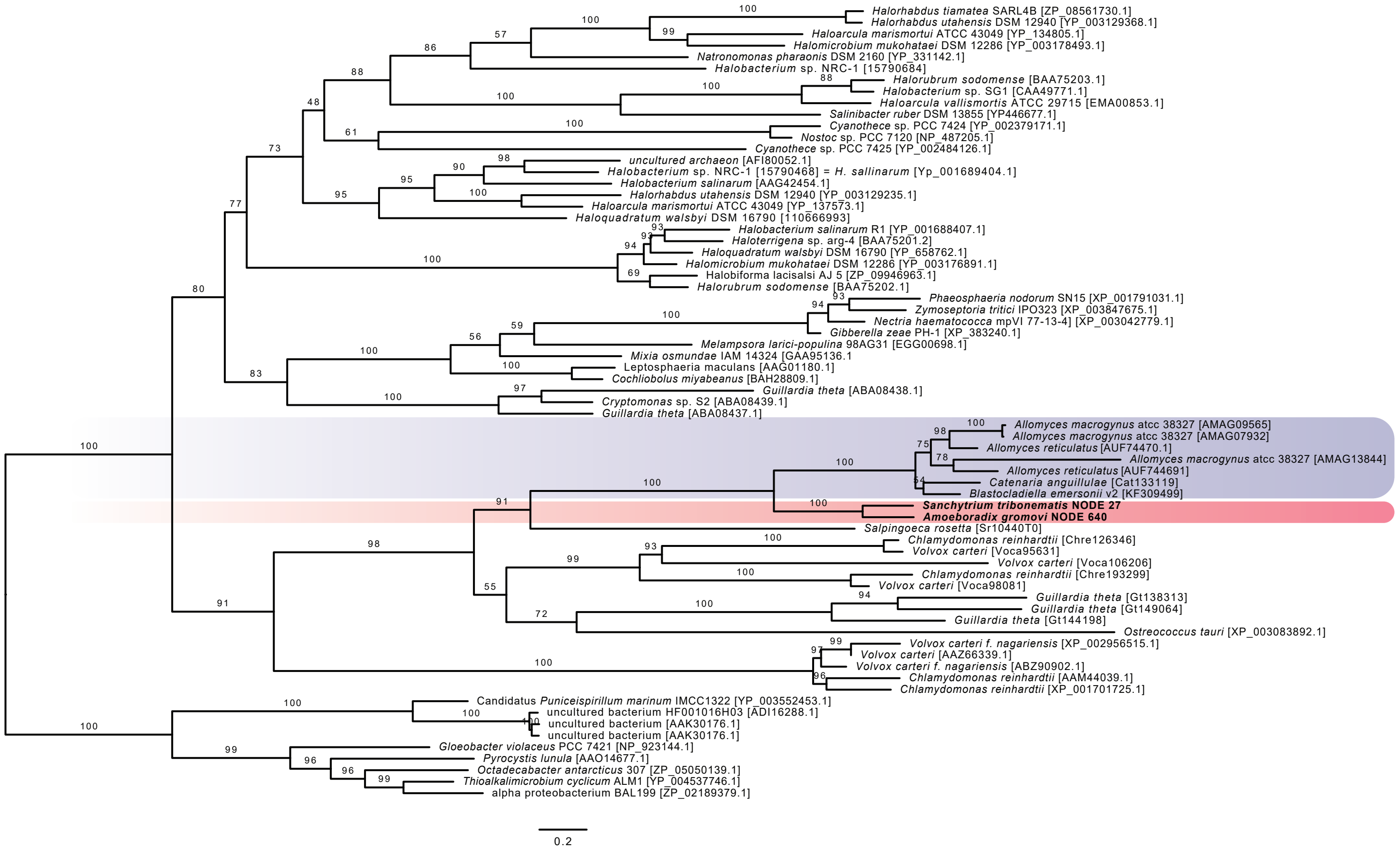

Supplementary Fig. 12. **a, Maximum likelihood tree based on the protein dataset of Avelar et al. (2014).** Reconstruction of the Type I rhodopsin domain of the BeGC1 gene-fusion, with 69 sequences and and 416 amino acid positions, it was inferred with IQ-TREE under the LG+F+I+G4 model and ultrafast bootstrap as statistical support.

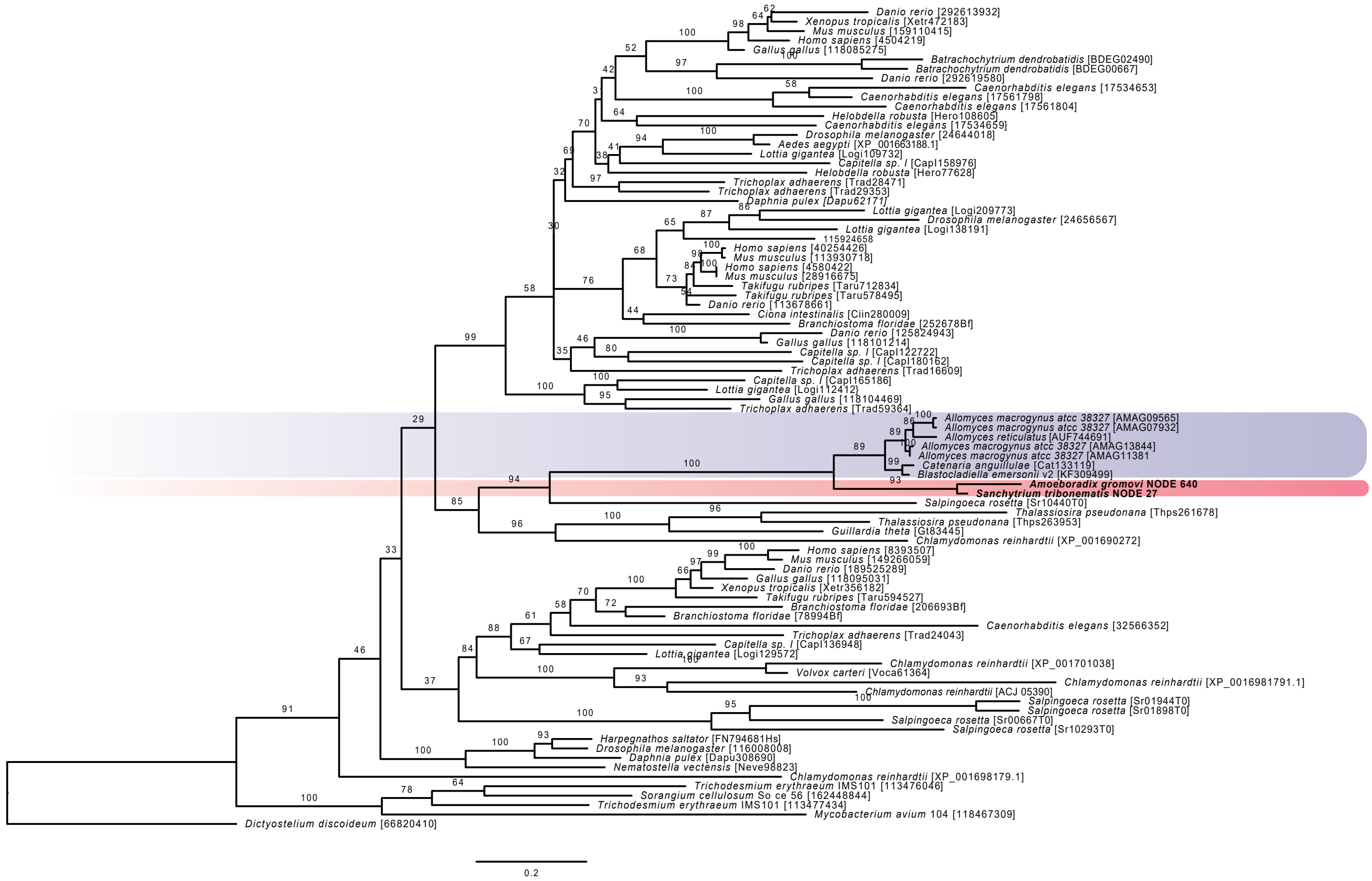

Supplementary Fig. 12. b, Maximum likelihood tree based on the protein dataset of Avelar et al. (2014). Reconstruction of the GC1 guanylyl-cyclase domain of the BeGC1 gene-fusion, with 87 sequences and 180 amino acid positions, it was inferred with IQ-TREE under the LG+G4 model and ultrafast bootstrap as statistical support.

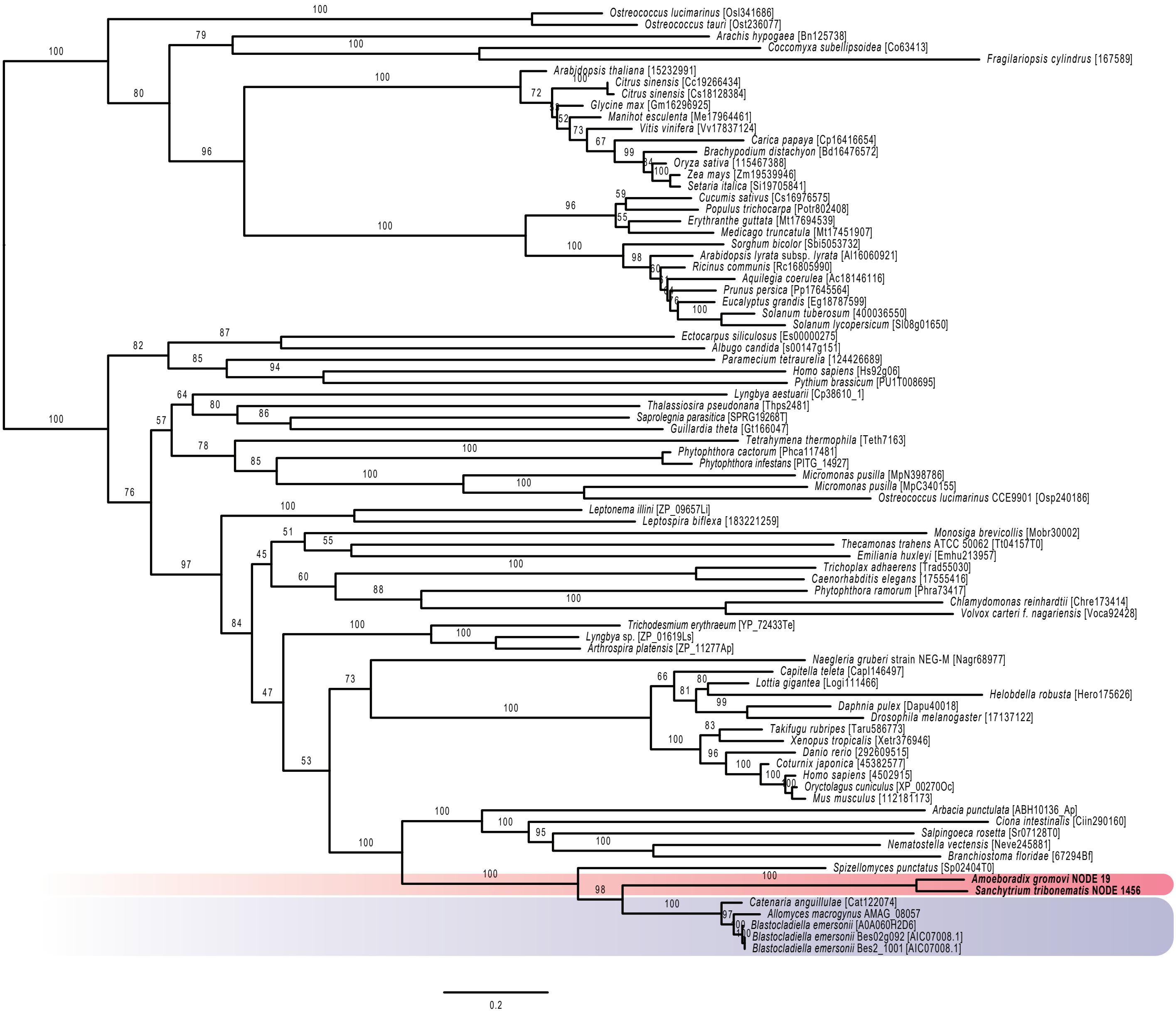

Supplementary Fig. 12. c, Maximum likelihood tree based on the protein dataset of Avelar et al. (2014). Reconstruction of the cyclic nucleotide gated channel BeCNG1, and 301 amino acid positions, it was inferred with IQ-TREE under the LG+F+I+G4 model and ultrafast bootstrap as statistical support.

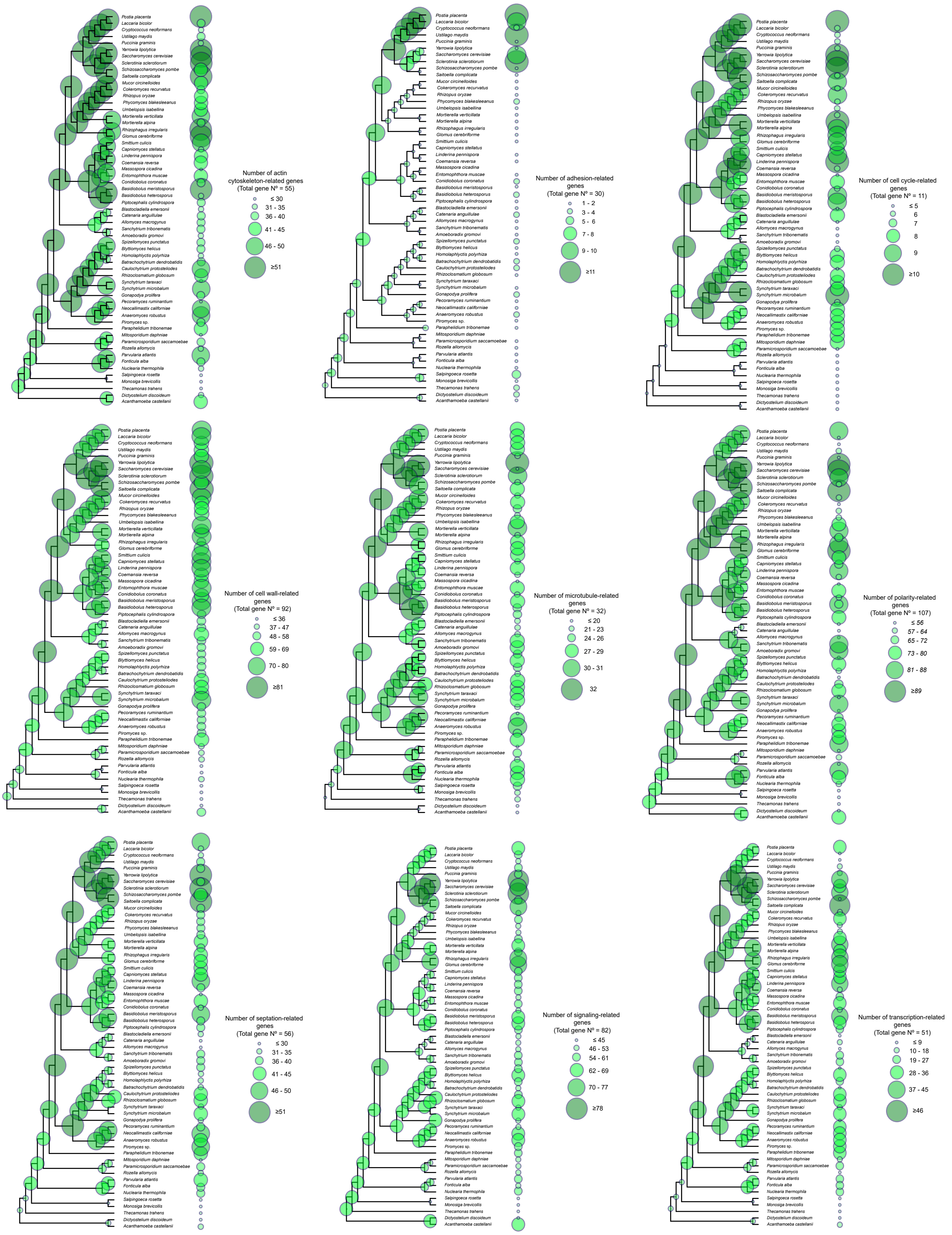

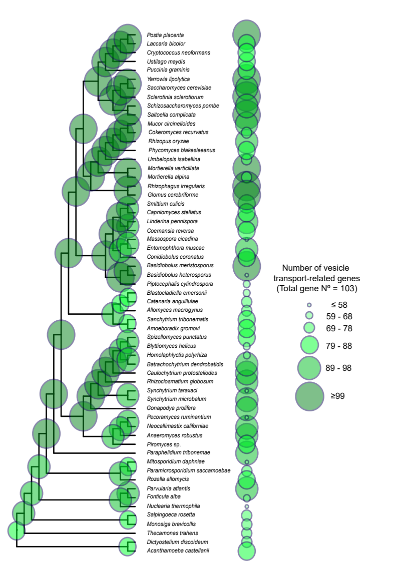

Supplementary Fig. 13. Evolution of hyphal multicellularity in Holomycota. Cladograms of Holomycota according to the GBE59 ML phylogenomic reconstruction for 10 different hyphal morphogenesis functional categories. Bubble size on the nodes and tips are represent the total number of reconstructed ancestral and extant hyphal multicellularity related gene proteins for each functional category.

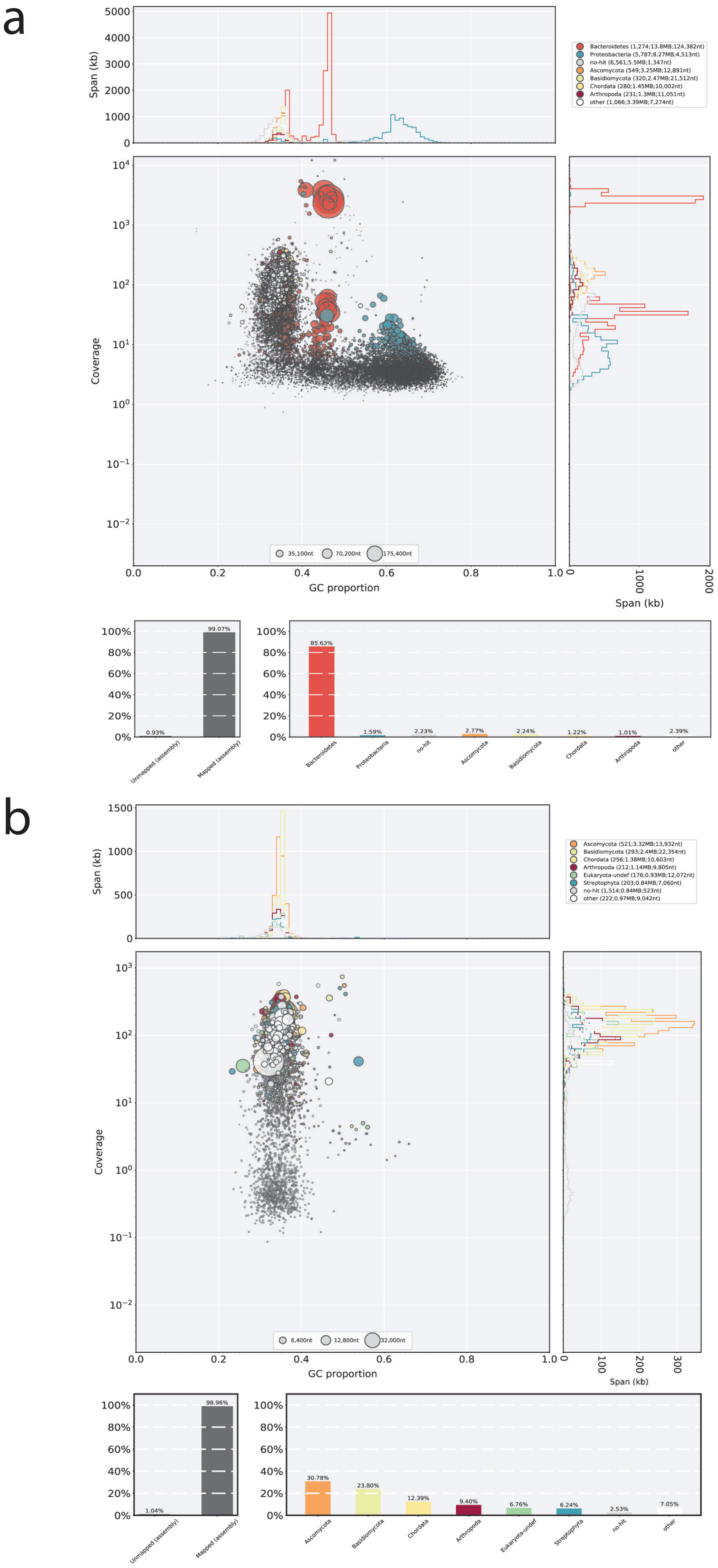

Supplementary Fig. 14. a-b, Blobplots showing the results before (**a**) and after (**b**) bacterial decontamination of the genome of *Sanchytrium tribonematis*. Taxon-annotated scatter plots decorated with coverage and GC content, the legend represents the taxonomic affiliation of sequences (phylum level) and lists count, total span and N50 by taxonomic group. Histograms show the proportion of reads of a library that are unmapped or mapped, showing the percentage of mapped reads by phylum.

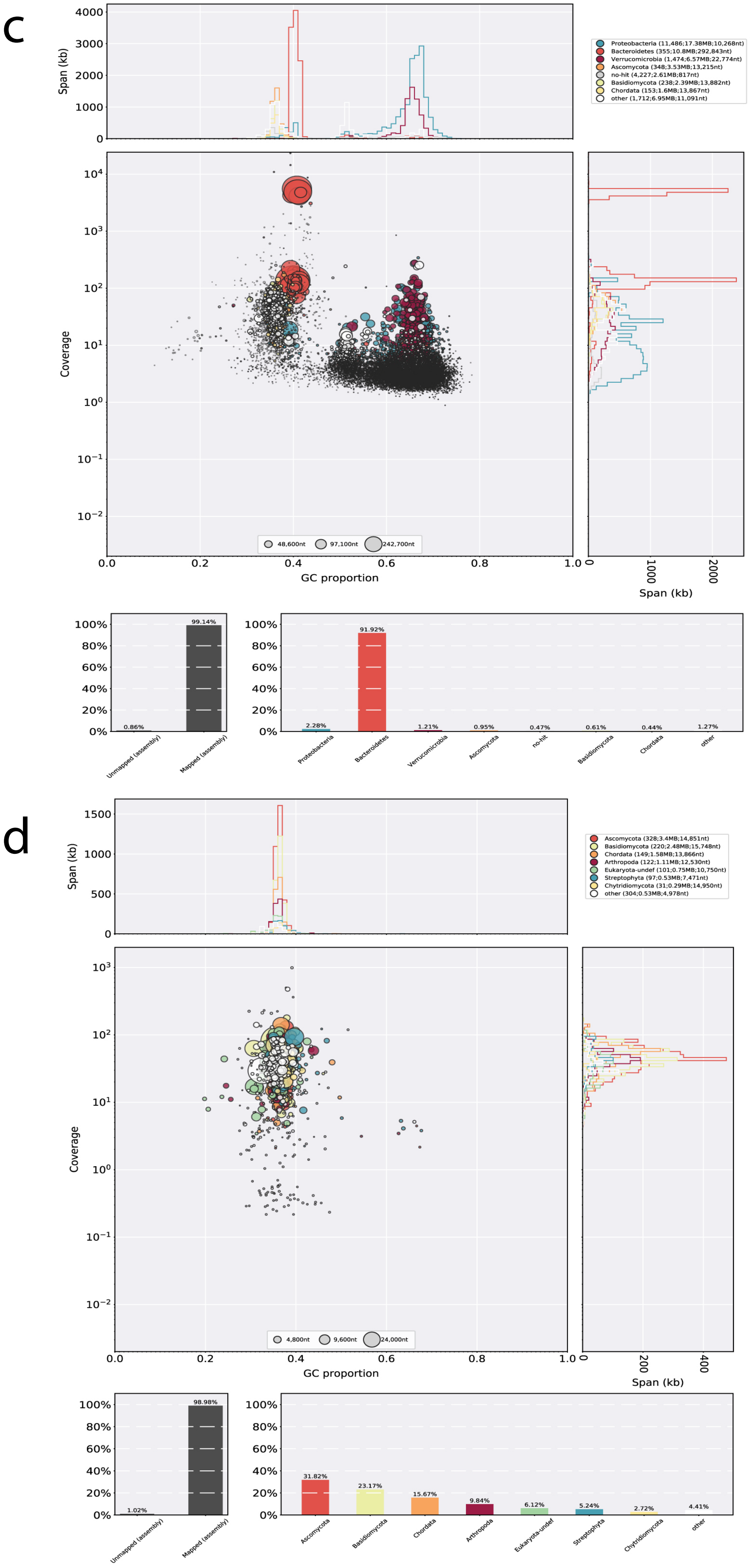

Supplementary Fig. 14. c-d, Blobplots showing the results before (**c**) and after (**d**) bacterial decontamination of the genome of *Amoeboradix gromovi*. Taxon-annotated scatter plots decorated with coverage and GC content, the legend represents the taxonomic affiliation of sequences (phylum level) and lists count, total span and N50 by taxonomic group. Histograms show the proportion of reads of a library that are unmapped or mapped, showing the percentage of mapped reads by phylum.
