# Supplementary material for: Phylogenomics of a new fungal phylum reveals multiple waves of reductive evolution across Holomycota
Source: Nat Commun. 2021 Aug 17;12:4973. doi: 10.1038/s41467-021-25308-w (PMC8371127; doi:10.1038/s41467-021-25308-w)
Supplement: Supplementary file 2 — Description of Additional Supplementary Files [file 41467_2021_25308_MOESM2_ESM.pdf]

Description of Additional Supplementary Files

File Name: Supplementary Data 1
Description: eggNOG annotation of the *Amoeboradix gromovi* predicted proteome.

File Name: Supplementary Data 2
Description: Proteins shared exclusively by *Amoeboradix gromovi* and *Sanchytrium tribonematis*.

File Name: Supplementary Data 3
Description: Alternative topology tests of phylogenomic analyses and removal of fast-evolving sites test. IQ-tree output alternative topology tests; and progressive exclusion of fastest evolving sites. B+F = Blastocladiomycota+Sanchytriaceae sister of all other fungi, C+F = Chytridiomycota sister of all other fungi, S+B = Sanchytriaceae within Blastocladiomycota, Dikarya = monophyly of Dikarya. Blue-shadowed cells indicate significance. The KH, SH and AU tests return p-values; a tree is rejected if its p-value < 0.05.

File Name: Supplementary Data 4
Description: Proteins involved in primary metabolism present in various eukaryotic genomes. Primary metabolism-related proteins are identified according to eggNOG classification.

File Name: Supplementary Data 5
Description: Carotenoid biosynthesis (beta-carotene) and cleavage enzymes found in sanchytrids, Blastocladiomycota and *Tribonema gayanum* (sanchytrid host), including the initial isoprenoid biosynthesis steps. NF: not found.

File Name: Supplementary Data 6
Description: Presence/absence (1/0) profiles of 619 proteins involved in hyphal morphogenesis in 69 eukaryotic proteomes. Based on the dataset of Kiss et al. (2019).

File Name: Supplementary Data 7
Description: Percentages of morphogenesis related genes predicted by COUNT (Csurös, 2010) for the CBZ (C: Chytridiomycota split, B: Blastocladiomycota + Sanchytriomycota split, Z: Zoopagomycota split) and NRA nodes (N: nucleariid split, R: Rozellida+Microsporidia split, A: Aphelida split), compared with the H node (Holozoa split).

File Name: Supplementary Data 8
Description: Origin of sequence data used in this work.
