# Supplementary material for: Phylogenomics of a new fungal phylum reveals multiple waves of reductive evolution across Holomycota
Source: Nat Commun. 2021 Aug 17;12:4973. doi: 10.1038/s41467-021-25308-w (PMC8371127; doi:10.1038/s41467-021-25308-w)
Supplement: Supplementary file 11 — Reporting summary [file 41467_2021_25308_MOESM11_ESM.pdf]

nature portfolio

Corresponding author(s): David Moreira

Last updated by author(s): Jun 21, 2021

# Reporting Summary

Nature Portfolio wishes to improve the reproducibility of the work that we publish. This form provides structure for consistency and transparency in reporting. For further information on Nature Portfolio policies, see our Editorial Policies and the Editorial Policy Checklist.

## Statistics

For all statistical analyses, confirm that the following items are present in the figure legend, table legend, main text, or Methods section.

| n/a | Confirmed | |
|---|---|---|
| ☐ | ☒ | The exact sample size (*n*) for each experimental group/condition, given as a discrete number and unit of measurement |
| ☒ | ☐ | A statement on whether measurements were taken from distinct samples or whether the same sample was measured repeatedly |
| ☒ | ☐ | The statistical test(s) used AND whether they are one- or two-sided<br>*Only common tests should be described solely by name; describe more complex techniques in the Methods section.* |
| ☒ | ☐ | A description of all covariates tested |
| ☒ | ☐ | A description of any assumptions or corrections, such as tests of normality and adjustment for multiple comparisons |
| ☒ | ☐ | A full description of the statistical parameters including central tendency (e.g. means) or other basic estimates (e.g. regression coefficient) AND variation (e.g. standard deviation) or associated estimates of uncertainty (e.g. confidence intervals) |
| ☐ | ☒ | For null hypothesis testing, the test statistic (e.g. *F*, *t*, *r*) with confidence intervals, effect sizes, degrees of freedom and *P* value noted<br>*Give P values as exact values whenever suitable.* |
| ☒ | ☐ | For Bayesian analysis, information on the choice of priors and Markov chain Monte Carlo settings |
| ☒ | ☐ | For hierarchical and complex designs, identification of the appropriate level for tests and full reporting of outcomes |
| ☐ | ☒ | Estimates of effect sizes (e.g. Cohen's *d*, Pearson's *r*), indicating how they were calculated |

*Our web collection on statistics for biologists contains articles on many of the points above.*

## Software and code

Policy information about availability of computer code

| | |
|---|---|
| Data collection | DNA and protein sequences of the species used in this study were downloaded from the GenBank public databases of nr [https://www.ncbi.nlm.nih.gov/nucleotide/ and https://www.ncbi.nlm.nih.gov/protein/], genome [https://www.ncbi.nlm.nih.gov/genome/], SRA [https://www.ncbi.nlm.nih.gov/sra/], the JGI genome database, CAZy database [cazy.org], [https://genome.jgi.doe.gov/portal/], and the mycoCLAP database [https://mycoclap.fungalgenomics.ca/mycoCLAP/] for more details see Supplementary Data 8. |

| Data analysis | Paired-end read quality: FastQC v0.11.9<br>Illumine adapter removal: Trimmomatic v0.32<br>Sequence assembly: SPAdes v3.9.1<br>Bacterial sequence removal: BlobTools v0.9.19<br>Open-reading frame translation: Transdecoder v2<br>Genome coverage estimation: QUAST v4.5 and Qualimap v2.2.1<br>Genome completeness and contamination estimation: BUSCO v2.0.1<br>Protein functional annotation: eggNOG mapper v2 and BlastKOALA v2.2<br>Carbohydrate-degrading enzymes annotation: mycoCLAP database v1 blast (https://mycoclap.fungalgenomics.ca/mycoCLAP/)<br>Mitochondrial genome annotation: MITOS v1<br>Sequence alignment: MAFFT v7<br>Alignment trimming: TrimAl v1.2<br>Concatenation of protein alignments: Geneious v6.0.6<br>Preliminary tree reconstruction: FastTree v2.1.7<br>Maximum likelihood phylogenetic tree reconstruction: IQ-TREE v1.6<br>Bayesian phylogenetic tree reconstruction: PhyloBayes-MPI v1.5<br>AU-tests: PHYLIP v3.695 and IQ-TREE v1.6<br>Gene gain/loss calculation: Count v10.04 |
|---|---|

For manuscripts utilizing custom algorithms or software that are central to the research but not yet described in published literature, software must be made available to editors and reviewers. We strongly encourage code deposition in a community repository (e.g. GitHub). See the Nature Portfolio guidelines for submitting code & software for further information.

## Data

Policy information about availability of data

All manuscripts must include a data availability statement. This statement should provide the following information, where applicable:

- Accession codes, unique identifiers, or web links for publicly available datasets
- A description of any restrictions on data availability
- For clinical datasets or third party data, please ensure that the statement adheres to our policy

The raw sequence data and assembled genomes generated in this work study have been deposited at the National Center for Biotechnology Information (NCBI) sequence databases under Bioprojects accession codes PRJNA668693 [https://www.ncbi.nlm.nih.gov/bioproject/PRJNA668693] and PRJNA668694 [https://www.ncbi.nlm.nih.gov/bioproject/PRJNA668694]. Additional data generated in this study (including alignments and phylogenetic trees) are available in the Figshare repository project 91439 at [https://figshare.com/projects/Sanchytriomycota_Galindo_etal/91439]. DNA and protein sequences of the species used in this study were downloaded from the GenBank public databases of nr [https://www.ncbi.nlm.nih.gov/nucleotide/ and https://www.ncbi.nlm.nih.gov/protein/], genome [https://www.ncbi.nlm.nih.gov/genome/], SRA [https://www.ncbi.nlm.nih.gov/sra/], the JGI genome database, CAZy database [cazy.org], [https://genome.jgi.doe.gov/portal/], and the mycoCLAP database [https://mycoclap.fungalgenomics.ca/mycoCLAP/] for more details see Supplementary Data 8.

# Field-specific reporting

Please select the one below that is the best fit for your research. If you are not sure, read the appropriate sections before making your selection.

☐ Life sciences ☐ Behavioural & social sciences ☒ Ecological, evolutionary & environmental sciences

For a reference copy of the document with all sections, see nature.com/documents/nr-reporting-summary-flat.pdf

# Ecological, evolutionary & environmental sciences study design

All studies must disclose on these points even when the disclosure is negative.

| Study description | In this study we determine the genome sequences of Sanchytrium tribonematis and Amoeboradix gromovi. Phylogenomic analyses using two large datasets and a rich taxon sampling of fungal species show that they occupy a key position in the fungal phylogeny as a deep-branching group sister to the Blastocladiomycota. Our trees also strongly support that the root of the tree of Fungi lies between the Chytridiomycota and all other Fungi. By combining this phylogenetic framework and the distribution of 60 flagellum-specific proteins, we infer three independent flagellum losses in Holomycota. We further propose that the residual flagellum of sanchytrids may function as a structural support for a lipid-based light sensing organelle homologous to the one found in Blastocladiomycota. Our new robust phylogenetic framework of Fungi also allows us to show that most of the hyphal morphogenesis genes had already evolved in unicellular lineages branching early in the holomycotan branch, revealing the importance of gene tinkering of multicellular Fungi. |
|---|---|
| Research sample | Sanchytrium tribonematis and Amoeboradix gromovi strains were obtained from the St. Petersburg Culture Collection (CALU). Sample choice was based on the avabilaity of these two unique strains of each species. The samples are supposed to represent a population of Sanchytrium tribonematis strain X-128 and Amoeboradix gromovi strain X-113. |
| Sampling strategy | Sanchytrium tribonematis and Amoeboradix gromovi sporangia were micromanipulated with an Eppendorf PatchMan NP2 micromanipulator using 19 µm VacuTip microcapillaries (Eppendorf) on an inverted Leica DlII3000 B microscope. Sporangia were separated from the algal host cells using a microblade mounted on the micromanipulator. No sample size calculation was performed, we considered that the large amount of DNA present in sporangia full of clonal cells was more than enough to perform MDA-amplification. This decision was confirmed to be right by the high BUSCO completeness values of our single-cell genomes. |

| | |
|---|---|
| Data collection | Sanchytrium tribonematis and Amoeboradix gromovi sporangia were micromanipulated with an Eppendorf PatchMan NP2 micromanipulator using 19 µm VacuTip microcapillaries (Eppendorf) on an inverted Leica Dlll3000 B microscope. Sporangia were separated from the algal host cells using a microblade mounted on the micromanipulator. Their genomes were MDA-amplified (by D.M. and P.L.G) and then sequenced using Illumina HiSeq 2500 v4 (Eurofins Genomics; Ebersberg, Germany). |
| Timing and spatial scale | Sanchytrium tribonematis and Amoeboradix gromovi sporangia were isolated after 2 weeks of growth, when we observed a maximum of hosts cells infected. For both species, a single culture of 10 ml was enough to obtain 10 sporangia. |
| Data exclusions | No data were excluded from the analyses. |
| Reproducibility | We carried out whole genome amplification with one sporangium of each species. In both cases, the DNA yield was enough to carry out Illumina sequencing. |
| Randomization | We did not carry out any statistical analysis that required randomization. Giving that neither our data nor the analyses performed were affected by any specific biases, randomization was not a required. |
| Blinding | We did not carry out any statistical analysis that required blinding. As with randomization giving that neither our data nor the analyses performed were affected by any specific biases, blinding was not a required |

Did the study involve field work? ☐ Yes ☒ No

# Reporting for specific materials, systems and methods

We require information from authors about some types of materials, experimental systems and methods used in many studies. Here, indicate whether each material, system or method listed is relevant to your study. If you are not sure if a list item applies to your research, read the appropriate section before selecting a response.

## Materials & experimental systems

| n/a | Involved in the study |
|---|---|
| ☒ | ☐ Antibodies |
| ☒ | ☐ Eukaryotic cell lines |
| ☒ | ☐ Palaeontology and archaeology |
| ☒ | ☐ Animals and other organisms |
| ☒ | ☐ Human research participants |
| ☒ | ☐ Clinical data |
| ☒ | ☐ Dual use research of concern |

## Methods

| n/a | Involved in the study |
|---|---|
| ☒ | ☐ ChIP-seq |
| ☒ | ☐ Flow cytometry |
| ☒ | ☐ MRI-based neuroimaging |
